# Nanocarrier of Pin1 inhibitor based on supercritical fluid technology inhibits cancer metastasis by blocking multiple signaling pathways

**DOI:** 10.1093/rb/rbad014

**Published:** 2023-02-27

**Authors:** Fengzhu Zhang, Aiwen Zhang, Youning Xie, Haiying Wen, Ranjith Kumar Kankala, Jing Huang, Anjun Zhang, Qi Wang, Biaoqi Chen, Haiyan Dong, Zhao Guo, Aizheng Chen, Dayun Yang

**Affiliations:** Fujian Key Laboratory of Translational Research in Cancer and Neurodegenerative Diseases, School of Basic Medical Sciences, Fujian Medical University, Fuzhou, 350108, PR China; Fujian Key Laboratory of Translational Research in Cancer and Neurodegenerative Diseases, School of Basic Medical Sciences, Fujian Medical University, Fuzhou, 350108, PR China; Fujian Key Laboratory of Translational Research in Cancer and Neurodegenerative Diseases, School of Basic Medical Sciences, Fujian Medical University, Fuzhou, 350108, PR China; Fujian Key Laboratory of Translational Research in Cancer and Neurodegenerative Diseases, School of Basic Medical Sciences, Fujian Medical University, Fuzhou, 350108, PR China; Institute of Biomaterials and Tissue Engineering, Huaqiao University, Xiamen, 361021, PR China; Fujian Provincial Key Laboratory of Biochemical Technology (Huaqiao University), Xiamen, 361021, PR China; Fujian Key Laboratory of Translational Research in Cancer and Neurodegenerative Diseases, School of Basic Medical Sciences, Fujian Medical University, Fuzhou, 350108, PR China; Fujian Key Laboratory of Translational Research in Cancer and Neurodegenerative Diseases, School of Basic Medical Sciences, Fujian Medical University, Fuzhou, 350108, PR China; Fujian Key Laboratory of Translational Research in Cancer and Neurodegenerative Diseases, School of Basic Medical Sciences, Fujian Medical University, Fuzhou, 350108, PR China; Institute of Biomaterials and Tissue Engineering, Huaqiao University, Xiamen, 361021, PR China; Fujian Provincial Key Laboratory of Biochemical Technology (Huaqiao University), Xiamen, 361021, PR China; Fujian Key Laboratory of Translational Research in Cancer and Neurodegenerative Diseases, School of Basic Medical Sciences, Fujian Medical University, Fuzhou, 350108, PR China; Fujian Key Laboratory of Translational Research in Cancer and Neurodegenerative Diseases, School of Basic Medical Sciences, Fujian Medical University, Fuzhou, 350108, PR China; Institute of Biomaterials and Tissue Engineering, Huaqiao University, Xiamen, 361021, PR China; Fujian Provincial Key Laboratory of Biochemical Technology (Huaqiao University), Xiamen, 361021, PR China; Fujian Key Laboratory of Translational Research in Cancer and Neurodegenerative Diseases, School of Basic Medical Sciences, Fujian Medical University, Fuzhou, 350108, PR China

**Keywords:** cancer metastasis, targeted therapy, Pin1 inhibitor, nano delivery, signaling pathway

## Abstract

Cancer metastasis is the primary cause of all cancer-related deaths due to the lack of effective targeted drugs that simultaneously block multiple signaling pathways that drive the dissemination and growth of cancer cells. The unique proline isomerase Pin1 activates numerous cancer pathways, but its role in cancer metastasis and the inhibitory efficacy of Pin1 inhibitors on cancer metastasis are unknown. Moreover, the applicability of Pin1 inhibitor―all-*trans* retinoic acid (ATRA) is limited due to its several drawbacks. Herein, uniform ATRA-loaded polylactic acid-polyethylene glycol block copolymer nanoparticles (ATRA-NPs) with high encapsulation efficiency, good cellular uptake, excellent controlled release performance and pharmacokinetics are developed using supercritical carbon dioxide processing combined with an optimized design. ATRA-NPs exhibited excellent biosafety and significant inhibition on the growth and metastasis of hepatocellular carcinoma. Pin1 played a key role in cancer metastasis and was the main target of ATRA-NPs. ATRA-NPs exerted their potent anti-metastatic effect by inhibiting Pin1 and then simultaneously blocking multiple signaling pathways and cancer epithelial–mesenchymal progression. Since ATRA-NPs could effectively couple the inhibition of cancer cell dissemination with cancer growth, it provided a novel therapeutic strategy for efficiently inhibiting cancer metastasis.

## Graphical Abstract

**Figure:**
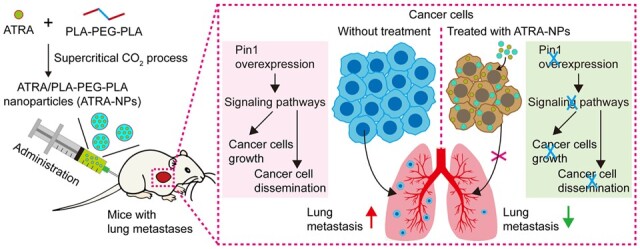

## Introduction

It is known that >90% of cancer deaths are caused by metastasis [1]. Hepatocellular carcinoma is a notable example, with a high recurrence and metastasis rate, leading to a low 5-year overall survival rate after current treatments include local surgery, systemic chemotherapy, targeted therapy and immunotherapy [2]. Hence, there is an urgent need to explore novel and practical approaches to treat cancer metastasis.

Typically, cancer metastasis comprises a sequence of events, including cancer cells leaving the initial tumor site, entering the bloodstream and surviving in circulation, extravasating at distant sites, reinitiating growth and establishing a metastatic tumor at the targeted site, which is regulated by a range of complex molecular changes [3–5]. Therefore, to effectively prevent and cure cancer metastasis, on the one hand, it is required to block signaling pathways triggering cancer cell dissemination in the initial stage. On the other hand, it is required to block cancer pathways driving cancer cell growth simultaneously.

Epithelial–mesenchymal transition (EMT) is a fundamental cellular process that plays vital roles in embryogenesis, tissue regeneration and tumor metastasis [6, 7]. In cancer, the activation of the EMT program is associated with various tumor-related functions, such as tumor invasion, tumor stemness and therapy resistance [8, 9]. Since EMT is involved in a variety of the hallmarks of cancer [10], it has become a promising target for the treatment of cancer metastasis. Hitherto, some natural or synthetic small molecules have been employed to inhibit cancer EMT by blocking signaling pathways, such as α-mangostin, curcumin and dexamethasone [11–13]. In addition, small interfering RNAs against EMT transcription factors or key EMT-related genes have been used to inhibit cancer EMT, including Zinc-finger E-box-binding 1 (ZEB1), twist family bHLH transcription factor 1 (TWIST1), Nogo-B receptor and Wnt family member 1 (WNT1) [14–17]. Although these strategies targeting EMT have shown promise in inhibiting cancer metastasis, none of them have been translated into clinical applications. The failure of their clinical application can be mainly attributed to two reasons. First, cancer EMT is a spectrum of transition states between the epithelial and mesenchymal phenotypes [18], regulated by multiple signaling pathways [6]. Predominantly, the heterogeneity between and within tumors further complicates EMT initiation and progression. Therefore, simply inhibiting an EMT-associated signaling pathway or deleting an EMT-related gene is often insufficient to inhibit the EMT process. Second, the reverse process of cancer EMT is necessary for the growth of metastatic cancer [19–21]. Although EMT inhibitors are highly effective in inhibiting cancer cell dissemination, they are not effective in inhibiting cancer cell growth. To effectively inhibit cancer metastasis, the inhibition of cancer cell dissemination should be coupled with the inhibition of cancer cell growth.

Typically, the phosphorylation of serine/threonine of proteins is a universal cellular signaling mechanism, and this phosphorylated protein is further regulated by isomerase Pin1 [22, 23]. The aberrant overexpression of Pin1 in various human tumors promotes tumorigenesis by activating a range of cancer pathways [22, 23]. Moreover, Pin1-null mice can develop normally but resist tumorigenesis [24–26]. Therefore, targeted inhibition of Pin1 will be able to inhibit a range of cancer pathways without causing obvious side effects [27]. Nevertheless, the role of Pin1 in cancer metastasis, especially the inhibitory effect of Pin1 inhibitors on cancer metastasis, is unknown.

**Figure 1.**
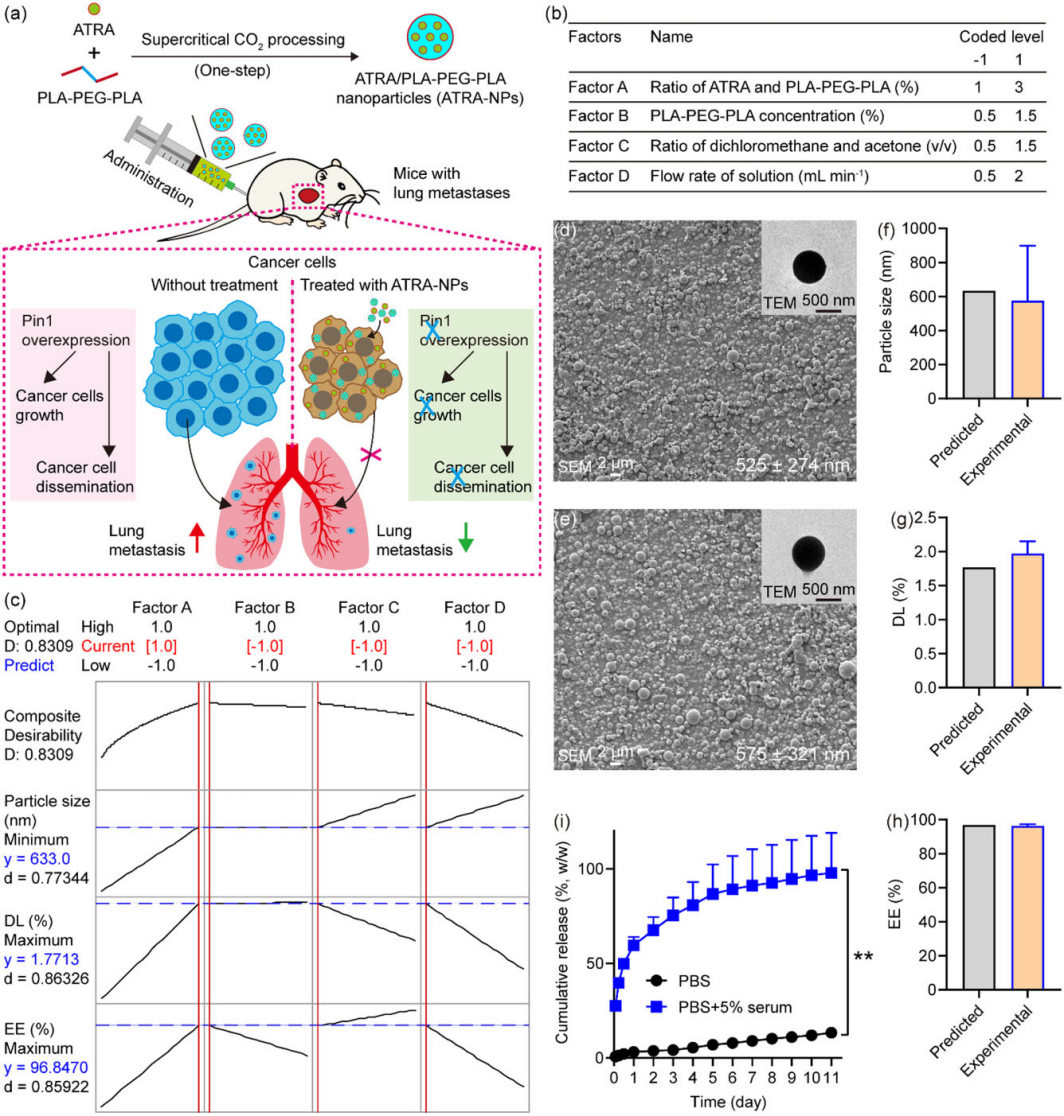
Preparation and characterization of ATRA-NPs. (**a**) Preparation and application of ATRA-NPs for inhibiting cancer growth and metastasis. (**b**) Experimental factors and levels. (**c**) Optimization analysis of the response of experimental factors. SEM and TEM images for (**d**) blank NPs and (**e**) ATRA-NPs. (**f**) Particle size, (**g**) DL and (**h**) EE of ATRA-NPs prepared under optimized experimental condition (*n* = 4). (**i**) Drug release of ATRA-NPs in PBS and PBS containing 5% FBS (*n* = 3).

All-*trans* retinoic acid (ATRA) is a first-line drug for the clinical treatment of acute promyelocytic leukemia [28], but its efficacy in the treatment of solid tumors is poor due to its various drawbacks, including short half-life, poor aqueous solubility, as well as easy inactivation by light, oxygen and heat. To overcome these limitations and enhance the anticancer efficacy of ATRA, some nano-formulations, including nano-sized liposomes, polymer nanocomposites and ATRA nanoparticles, have been prepared for delivering ATRA to tumor tissues [29–32]. Despite the success in offering improved efficacy, these preparation strategies suffer from various limitations of time-consuming and require additional steps for organic solvent removal, as well as product drying, affecting the anti-tumor activity of ATRA, which could be the reasons for unsuccessful clinical translation [33]. Compared with these traditional methods, supercritical carbon dioxide (CO_2_) fluid technology offers several advantages, such as operating under an inert gas environment and mild temperature conditions and obtaining dry products without organic solvents in one step [34]. Since polylactic acid (PLA)–polyethylene glycol (PEG) block copolymers have the advantages of both PLA and PEG, including their biodegradability, good biocompatibility, and amphiphilic characteristics, they have been used for drug delivery [35]. Motivated by these aspects, Herein, we developed ATRA-loaded PLA–PEG block copolymer nanoparticles (ATRA-NPs) by a one-step method using the supercritical CO_2_ process, and then further investigated the inhibitory efficacy and mechanism of ATRA-NPs on cancer metastasis (Fig. 1a). ATRA-NPs are expected to couple the inhibition of cancer cell dissemination with the inhibition of cancer cell growth, thereby providing a novel therapeutic strategy for efficiently inhibiting cancer metastasis.

## Materials and methods

### Materials, cells and animals

ATRA, acitretin, fluorescein isothiocyanate isomer I (FITC) and corn oil were acquired from Sigma-Aldrich (Missouri, USA). The 21-day ATRA sustained-release pellet was obtained from Innovative Research of America (Florida, USA). PLA–PEG–PLA triblock polymers (Mw 110 kDa, PEG 9%) were obtained from Jinan Daigang Biomaterial (Shandong, China). Cell lysis buffer for IP, BCA protein assay kit, protease inhibitor cocktail, 4′, 6-diamidino-2-phenylindole (DAPI) kit, hematoxylin and eosin (H&E) kit, crystal violet staining solution and citrate–EDTA antigen retrieval solution were procured from Beyotime Biotechnology (Shanghai, China).

Hepatocellular carcinoma cell lines were provided by the Cell Bank (Shanghai, China). Cells were cultured with Dulbecco’s Modified Eagle Medium (DMEM; ThermoFisher, USA) containing 10% fetal bovine serum (FBS; PAN, Germany) and 1% penicillin–streptomycin solution (HyClone, USA) at 37°C under 5% CO_2_ in a humidified incubator. Male BALB/c nu/nu mice were obtained from Shanghai Laboratory Animal Center (SLAC, Shanghai, China). The animal care and experimental protocols were performed in accordance with and approved by the Experimental Animal Ethics Committee of Fujian Medical University.

### Preparation and characterizations

Blank NPs, ATRA-NPs and FITC-NPs were fabricated by the supercritical anti-solvent method according to previously reported procedure with modifications [36]. Briefly, ATRA and PLA–PEG–PLA were dissolved in dichloromethane (DCM) and mixed with acetone, and then injected into high-pressure vessel with supercritical CO_2_ to obtain dried nanoparticles. According to the results of our pilot experiments, pressure, temperature and CO_2_ flow rate in the processing were 8.0 MPa, 45°C and 35 g min^−1^, respectively. The blank NPs and FITC-NPs were also prepared using this condition. To evaluate the influence of four key factors, the ratio of ATRA and PLA–PEG–PLA (Factor A), PLA–PEG–PLA concentration (Factor B), the ratio of DCM and acetone (Factor C) and flow rate of solution (Factor D), on ATRA-NPs properties, a 2^4^ factorial experiment was designed following parameters shown in Fig. 1b. ATRA-NPs were fabricated under 16 conditions, and the influences of four factors on particle size and drug loading (DL), as well as encapsulation efficiency (EE) were systematically analyzed. Furthermore, optimized experimental condition for preparing ATRA-NPs with minimum particle size, maximum DL and EE was predicted using Minitab’s Response Optimizer. Moreover, ATRA-NPs were further prepared following the predicted condition, and their particle size, DL and EE were assayed and compared with the predicted values. The morphology and structure of particles were examined by scanning electron microscope (SEM; QUANTA450, FEI) and transmission electron microscope (TEM; H7650, HITACHI). The particle size was obtained by analyzing the SEM images of the sample using SmileView software. For each sample, the sizes of at least 400 particles were measured.

### DL, EE and drug release assays

The DL, EE and drug release of ATRA-NPs were determined according to previously reported procedure with some modifications [27]. To determine the unencapsulated ATRA, ATRA-NPs were washed with 50% methanol and filtrated with 20 nm membrane, and then analyzed using a microplate reader at 360 nm and calculated with a standard curve of ATRA solution. The drug release behavior of ATRA-NPs was measured in phosphate-buffered saline (PBS; HyClone, USA) with or without FBS.

### Cell uptake and cancer cell growth assays

The cell uptake, cell proliferation and cell focus formation assays were performed according to previously reported procedure with modifications [27]. Briefly, for cell uptake assay, HuH7 cells were treated with FITC-NPs (0.13 mg ml^−1^) for 4 and 24 h. After that, the cells were fixed and stained with DAPI, and finally observed under a Zeiss microscope. For cell proliferation assay, HuH7 (3 × 10^3^) and PLC (6 × 10^3^) cells were seeded in 96-well plates. After treating with blank NPs, ATRA or ATRA-NPs for 72 h, the cell viability was assayed using thiazolyl blue tetrazolium bromide (MTT; Sangon, China). The half maximal inhibitory concentration (IC_50_) value of the drug was analyzed using SPSS 20.0 software. For cell focus formation assay, HuH7 (1 × 10^3^) cells were seeded in six-well plates. After treating with ATRA and ATRA-NPs for 72 h, the cells were cultured with fresh medium for another 14 days. Finally, the cell foci were visualized with crystal violet staining and quantified with Image software.

### Production of stable gene knockdown or overexpression cell lines

Pin1 knockdown cells were established according to previously reported procedure [27]. In brief, cells were infected with lentiviruses carrying *PIN1* shRNA (named shPin1) or scrambled shRNA (named shV), and then selected with puromycin. HuH7 cells expressing luciferase (named HuH7-Luc) were also established by lentivirus infection, then selected by puromycin and validated by bioluminescence imaging analysis (IVIS spectrum imaging system, PerkinElmer, USA).

### Cell migration

HuH7 (5 × 10^4^), HuH7-shV (5 × 10^4^), HuH7-shPin1 (5 × 10^4^), HepG2 (1 × 10^5^), HepG2-shV (1 × 10^5^) or HepG2-shPin1 (1 × 10^5^) in 200 µl of medium without serum but containing ATRA or ATRA-NPs was added to the upper chamber (24-well inserts, Millipore). Meanwhile, 500 µl of medium with 10% FBS was added to the lower chamber. After 72 h of culture, nonmigrated cells on the upper surface of the membrane were removed by wiping them with a cotton swab. After washing with PBS thrice, the membrane was fixed with methanol and stained with crystal violet. The migrated cells per chamber were observed using a Zeiss microscope and quantified by Image software.

### Cell invasion

Cell invasion assay was performed according to the above procedure of cell migration assay with a few modifications. In brief, 3 × 10^5^ HuH7, HuH7-shV, HuH7-shPin1, HepG2, HepG2-shV or HepG2-shPin1 in 200 µl of medium without serum but containing ATRA or ATRA-NPs was added to the upper chamber coated with the Matrigel matrix (24-well inserts, BD Biocoat). After 72 h of culture, the invaded cells were evaluated.

### Wound healing

HuH7 (5 × 10^5^) or HepG2 (8 × 10^5^) was seeded on six-well plates and cultured normally overnight. After that, cells were starved for 12 h in a serum-free medium. Further, linear wounds were scratched through the cell layer by a sterile pipette tip, and the cells were cultured with medium without serum but containing ATRA or ATRA-NPs for 72 h. The wound healing was observed using microscope and measured by Image software.

### Western blot

Total proteins were extracted using IP lysis buffer and quantified with BCA assay. Then, proteins were separated by sodium dodecyl sulfate–polyacrylamide gel electrophoresis and transferred onto a polyvinylidene difluoride membrane. Further, the membrane was blocked with 5% nonfat dry milk solution in tris-buffered saline with tween (TBST) for 1 h at room temperature and then incubated with primary antibodies overnight at 4°C. After washing with TBST, the membrane was incubated with the secondary antibodies for 1–2 h at room temperature. The targeted protein expression was detected by chemiluminescence. β-Actin was used as an internal reference protein. The primary antibodies (1:1000) used were as follows: PIN1 (Proteintech, China); E-cadherin, Vimentin and β-catenin (CST, USA); N-cadherin, matrix metallopeptidase 2 (MMP2), snail family transcriptional repressor 1 (SNAIL), RAF proto-oncogene serine/threonine-protein kinase (RAF-1), dual specificity mitogen-activated protein kinase kinase 1/2 (MEK 1/2), mitogen-activated protein kinase 3 (ERK 1/2), phosphatidylinositol 3-kinase (PI3K), protein kinase B (AKT), nuclear factor kappa-B (NF-κB), notch receptor 1 (NOTCH1), signal transducer and activator of transcription 3 (STAT3) and hypoxia-inducible factor 1-alpha (HIF1-α) (ImmunoWay, USA).

### Pharmacokinetic and biodistribution studies

The pharmacokinetics of ATRA-NPs were evaluated in 8-week-old mice according to previously reported procedure with modifications [27]. Briefly, ATRA-NPs were dispersed in saline and intraperitoneally injected into mice at a dose of 15 mg kg^−1^. The 21-day ATRA sustained-release pellets (as a control group) were implanted subcutaneously in the neck of mice. Each group contained four mice at each experimental time. After 0.5, 2, 4, 8, 24 and 48 h of administration, the blood of mice was collected and centrifuged to obtain plasma samples. The ATRA amount in plasma was measured using liquid chromatography–tandem mass spectrometry. Various pharmacokinetic parameters were analyzed using DAS 3.2.3 pharmacokinetic software.

For biodistribution studies, ATRA-NPs were injected intraperitoneally into mice at a dose of 15 mg kg^−1^. After 4 and 24 h of administration, the mice were sacrificed, major organs were collected and homogenized in saline (3 µl saline/1 mg tissue). A 4-fold volume of methanol was added to this tissue homogenate, then vortexed for 5 min, followed by centrifugation at 10 000 rpm for 10 min at 4°C. The ATRA content in this methanol was determined by the above method.

### *In vivo* anti-metastasis studies

On Day 0, 2.5 × 10^6^ HuH7 cells were intravenously injected into mice, which were then randomly divided into four groups of control (saline), ATRA, ATRA pellet and ATRA-NPs. In addition, normal mice without cell injection were used as control. Moreover, HuH7-Luc cells were also injected intravenously into mice. On Day 7 after injection of the HuH7-Luc cells, the *in vivo* bioluminescence imaging of mice was analyzed using IVIS spectrum imaging system (PerkinElmer, USA). The dose of D-luciferin (APExBIO, USA) was 150 mg kg^−1^. From Day 7, ATRA and ATRA-NPs were administered intraperitoneally at a dose of 15 mg kg^−1^ twice a week for 3 weeks. The 21-day ATRA sustained-release pellet was implanted subcutaneously in the neck of mice. Mice body weight was recorded weekly. On Day 56, the mice were sacrificed, and major organs and blood samples were collected.

### Histology and immunohistofluorescence

The collected organs were embedded in paraffin, sliced, stained routinely with H&E and examined, as well as photographed using a Zeiss microscope. To quantify the lung metastatic burden, the metastatic nodules per lung were counted. For immunohistofluorescence analysis, the lung tissue sections with a thickness of 5 µm were deparaffinized and then repaired with citrate–EDTA antigen retrieval solution. Afterward, lung tissue sections were blocked using FBS containing 0.03% Triton X-100 and then incubated with primary antibodies containing 0.1% Tween 20 for 24 h at 4°C. After washing, lung tissue sections were incubated with secondary antibodies and DAPI solution. After mounting with a mounting medium, all sections were examined under a fluorescence microscope. Information on primary antibodies (1:400) was as follows: PIN1 (Proteintech, China); E-cadherin and Vimentin (CST, USA); N-cadherin, MMP2, SNAIL and HIF1-α (ImmunoWay, USA). Information on secondary antibodies was as follows: Alexa Fluor 488/594-conjugated goat anti-rabbit IgG (Invitrogen, USA).

### Blood biochemical analysis

The collected blood samples were centrifuged to obtain plasma, and then the level of functional indicators of the heart, liver and kidney in the plasma was determined using Roche Cobas 8000 modular analyzer series.

### Statistical analysis

Statistical analyses were carried out with SPSS 20.0 software. Data were presented as mean ± standard deviation. Differences between groups were measured using *t*-tests or one-way analysis of variance. All significant values were indicated as follows: *P <* *0.05, **P <* *0.01, ***P <* *0.001.

## Results and discussion

### Preparation and characterizations of ATRA-NPs

ATRA-NPs were prepared by a one-step supercritical CO_2_ process. Since the experimental factors have significant effects on the properties of drug-loaded microspheres [37, 38], we systematically investigated the effects of four key experimental factors on the performance of ATRA-NPs by a full factorial design (Fig. 1b). It was observed from the SEM results that ATRA-NPs prepared under 16 different experimental conditions showed excellent spherical morphologies (Supplementary Fig. S1). However, the particle sizes varied from 372 nm to 1524 nm (Supplementary Table S1). Further, the DL and EE of ATRA-NPs were in the range of 0.1–2% and 77.6–100%, respectively (Supplementary Table S1).

To determine the importance of experimental factors and their effects on the particle size, DL and EE of ATRA-NPs, the experimental results from the full factorial design were analyzed. Although the effects of the four experimental factors on the particle size of ATRA-NPs showed no significance, the particle size of ATRA-NPs increased with the increase of experimental factor level (Supplementary Fig. S2). Factor A significantly affected the DL of ATRA-NPs (Supplementary Fig. S3a). Moreover, the DL increased with Factor A or Factor B but decreased with Factor D (Supplementary Fig. S3b). Although the four experimental factors had no significant effects on the EE of ATRA-NPs, the EE increased with Factor A, Factor C or Factor D (Supplementary Fig. S4). From the above analyses, it was observed that the four experimental factors showed complex effects on the performance of ATRA-NPs.

To obtain the optimum experimental conditions for preparing ATRA-NPs with minimum particle size, maximum DL and EE, a response optimization analysis was further performed. As shown in Fig. 1c, the optimum experimental conditions predicted for the preparation of ATRA-NPs with minimum particle size, maximum DL and EE were as follows: Factor A = 3%, Factor B = 0.5%, Factor C = 0.5 and Factor D = 0.5 ml min^−1^. The predicted particle size, DL and EE of ATRA-NPs prepared under this condition were 633 nm, 1.8% and 96.8%, respectively (Fig. 1c). To verify the predicted values, the morphology, particle size, DL and EE of ATRA-NPs prepared under the predicted experimental condition were further evaluated. The experimental validation results showed that the prepared blank NPs and ATRA-NPs displayed fairly good spherical morphology and smooth surface (Fig. 1d and e). Moreover, the TEM results showed that the blank NPs and ATRA-NPs had compact structures, indicating they are solid spheres (Fig. 1d and e). The particle size, DL and EE of ATRA-NPs were 575 ± 321 nm, 2.0 ± 0.2% and 96.4 ± 1.1%, respectively (Fig. 1f–h). These results confirmed that ideal ATRA-NPs with excellent properties could be prepared under these experimental conditions.

To understand the drug release behavior of ATRA-NPs, the cumulative release rate of ATRA-NPs in PBS and PBS-containing serum was recorded. As shown in Fig. 1i, ATRA release from ATRA-NPs in both media increased with time. Simultaneously, a significant difference in ATRA release rates in the two media was noted. In PBS, the ATRA-NPs released only 3.1% of the loaded ATRA within the first day and 13.3% within 11 days. In contrast, the cumulative release rate of ATRA in serum-containing PBS was 59.5% on Day 1 and 97.8% on Day 11. Anyway, this result indicated that ATRA-NPs could slowly release ATRA even in the medium containing serum.

### Cellular uptake and *in vitro* anti-tumor growth

**Figure 2.**
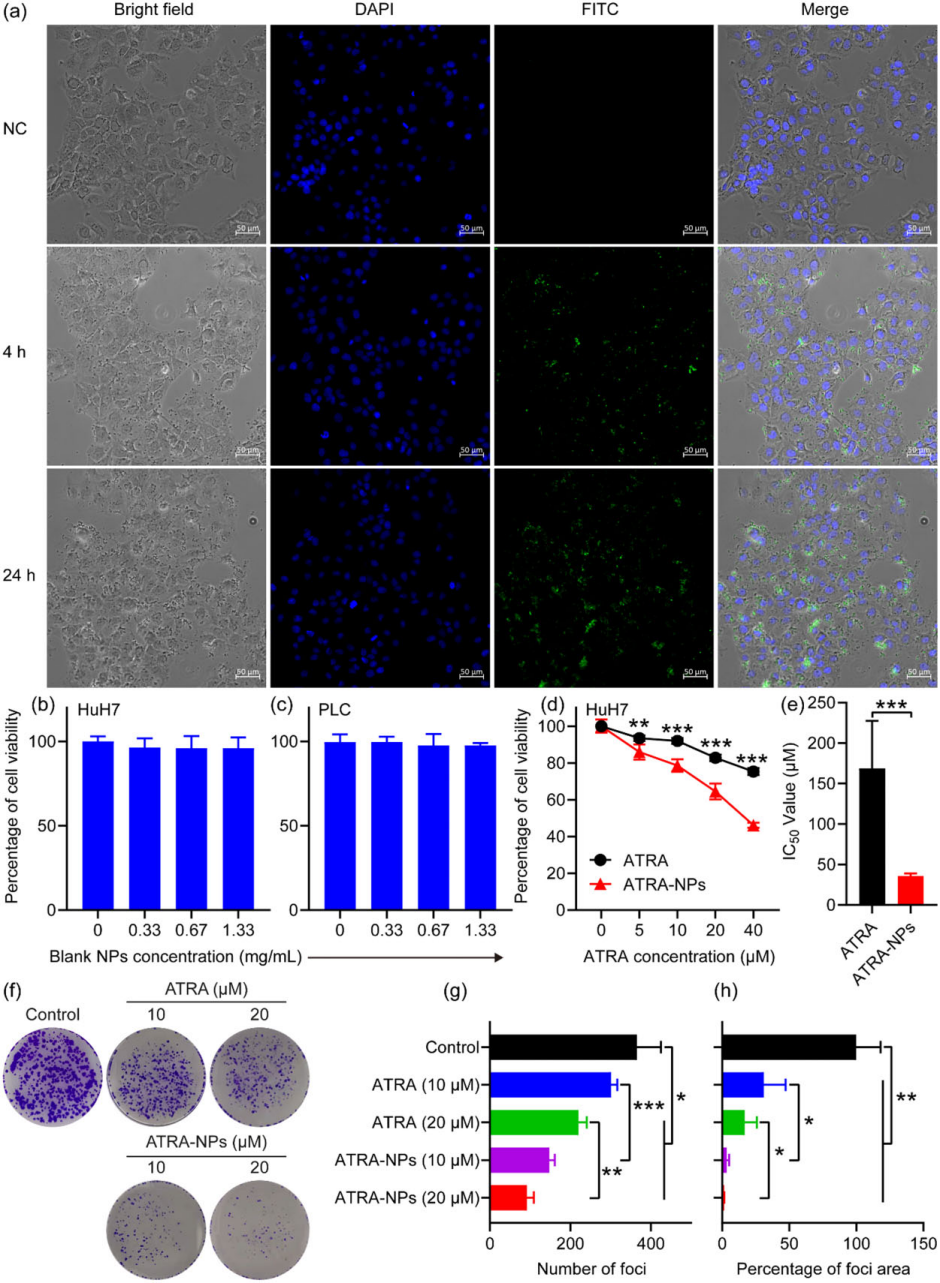
Cellular uptake and *in vitro* anti-tumor growth. (**a**) Cellular uptake of FITC-NPs in HuH7 cells. Viability of HuH7 cells (**b**) and PLC cells (**c**) after 72 h treatment with blank NPs (*n* = 6). The concentrations of blank NPs at 0.33, 0.67 and 1.33 mg ml^−1^ correspond to the concentrations of ATRA-NPs containing 20, 40 and 80 µM ATRA. (**d**) Effects of ATRA and ATRA-NPs on HuH7 cell viability after 72 h of treatment (*n* = 6). (**e**) Corresponding IC_50_ values. (**f**–**h**) Effects of ATRA and ATRA-NPs on HuH7 cell focus formation (*n* = 3).

To understand the biodistribution of our nanoparticles in cells, cellular uptake of FITC-NPs by HuH7 cells was investigated. After 4 h of incubation, the cells in the treatment group were the same as in the control. However, numerous green dots and a few green spots were observed in the proximity of the nucleus (Fig. 2a), suggesting that FITC-NPs were internalized through the cytoplasm. Compared with the control group, the black dots in the cells of the treatment group increased with time. At the same time, many green spots were observed around the nucleus (Fig. 2a). Together, this result demonstrated that ATRA-NPs could be internalized by cells, which was consistent with the previous findings [39, 40].

To determine the efficacy of ATRA-NPs against tumor cell growth, we examined the effects of the nanoparticles on the viability of hepatocellular carcinoma cells. As anticipated, the blank NPs almost had no inhibition on the HuH7 and PLC cells even at 1.33 mg ml^−1^ (corresponding to the concentration of ATRA-NPs containing 80 µM ATRA; Fig. 2b and c), demonstrating that the blank NPs showed excellent biocompatibility. In the cases of ATRA and ATRA-NPs, they generated a dose-dependent inhibition on HuH7 cells after 72 h of treatment. Compared with ATRA, ATRA-NPs generated a significant inhibitory effect on Huh7 cells (Fig. 2d), and the corresponding IC_50_ significantly decreased by about 4.7-fold (Fig. 2e). This demonstrated that the ATRA-NPs could significantly augment the efficacy of ATRA against cancer growth and reduce its dose. To further determine the inhibition of ATRA-NPs on cancer growth, the inhibition of the nanoparticles on cancer cell focus formation was investigated. As shown in Fig. 2f–h, after treating with ATRA and ATRA-NPs for 72 h, the focus formation of HuH7 cells decreased significantly in a dose-dependent manner. Moreover, the inhibitory effect of ATRA-NPs on cancer cell focus formation was significantly higher than that of ATRA (Fig. 2f–h). Overall, these results demonstrated that ATRA-NPs could significantly augment the inhibitory effect of ATRA on cancer growth. In addition, the nanoparticle itself showed excellent biocompatibility. ATRA-NPs could not only release ATRA slowly, but also be internalized by cancer cells, so it could overcome the many shortcomings of ATRA, such as short half-life. This explains why ATRA-NPs could significantly enhance the anti-tumor effects of ATRA.

### *In vitro* anti-metastasis

**Figure 3.**
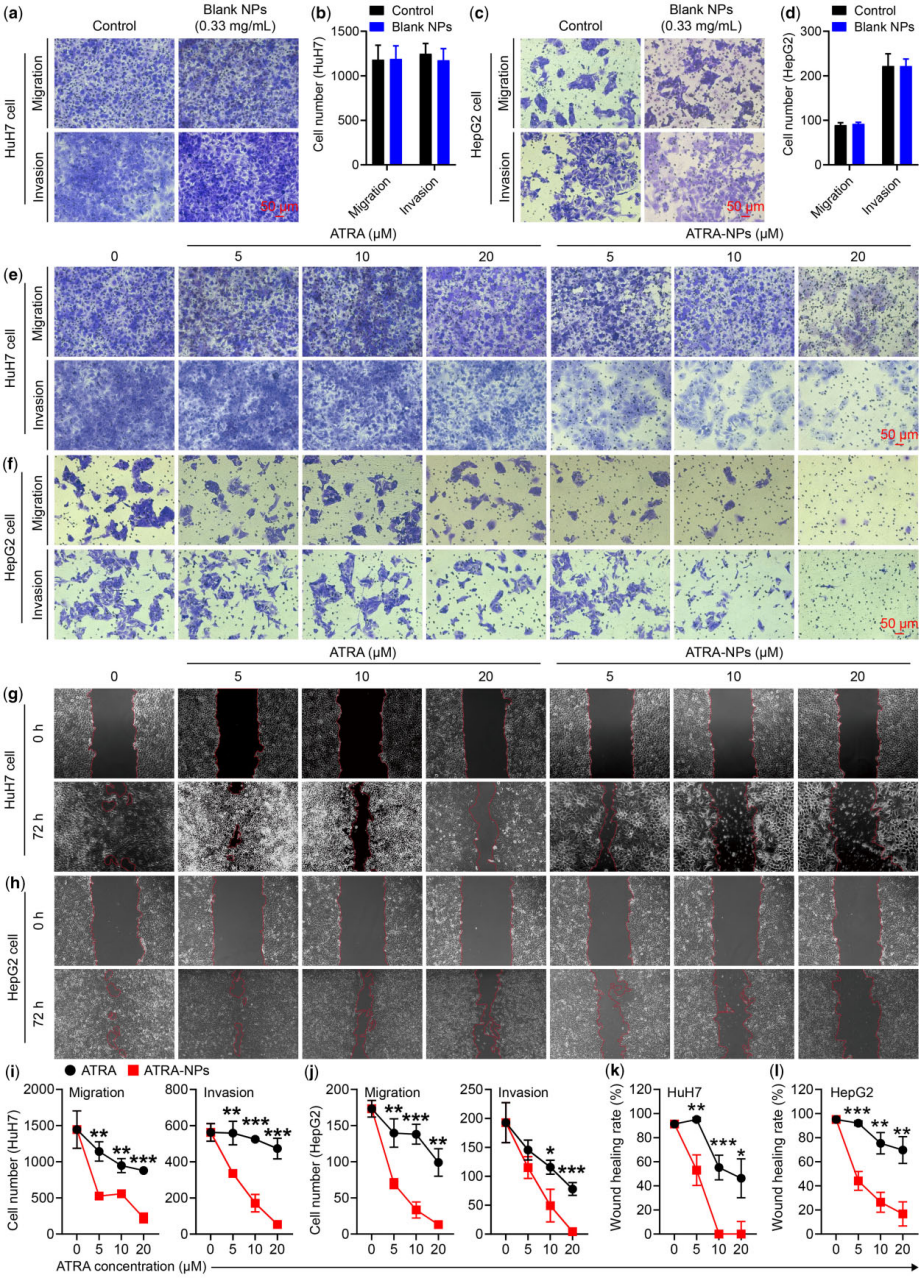
*In vitro* anti-tumor metastasis. (**a**) Images and (**b**) numbers of migrated and invaded HuH7 cells after 72 h treatment with 0.33 mg ml^−1^ blank NPs (*n* = 3). (**c**) Images and (**d**) numbers of migrated and invaded HepG2 cells after 72 h treatment with 0.33 mg ml^−1^ blank NPs (*n* = 3). (**e**) Images and (**i**) numbers of migrated and invaded HuH7 cells after 72 h of treatment (*n* = 3). (**f**) Images and (**j**) numbers of migrated and invaded HepG2 cells after 72 h of treatment (*n* = 3). (**g**) Images and (**k**) quantitative analysis of wound healing of HuH7 cells after 72 h of treatment (*n* = 3). (**h**) Images and (**l**) quantitative analysis of wound healing of HepG2 cells after 72 h of treatment (*n* = 3).

Because human liver tumor cell lines HuH7 and HepG2 have high expression of Pin1 and are widely used in liver tumor growth and metastasis studies, we selected these two cell lines as model cells to investigate the anti-metastasis effect of ATRA-NPs *in vitro*. To determine the anti-metastasis efficacy of ATRA-NPs, the inhibitory effects of the nanoparticles on cancer cell migration and invasion were investigated. As shown in Fig. 3a and b, the blank NPs almost had no inhibitory effect on the migration and invasion of HuH7 cells. In contrast, ATRA and ATRA-NPs generated dose-dependent inhibitory effects on the migration and invasion of HuH7 cells (Fig. 3e and i). The migrated HuH7 cells in the ATRA-NPs-treated group at a concentration range of 5, 10 and 20 µM was 526 ± 12, 557 ± 35 and 223 ± 68, respectively, which were very significantly lesser than that in the ATRA-treated group (Fig. 3i). Moreover, the invaded HuH7 cells in the ATRA-NPs-treated group at a concentration range of 5, 10 and 20 µM was 335 ± 10, 171 ± 48 and 54 ± 9, respectively, which were very significantly lesser than that in the ATRA-treated group (Fig. 3i). Similar inhibitory behaviors of the nanoparticles on cell migration and invasion were detected in HepG2 cells. As depicted in Fig. 3c and d, the blank NPs presented no inhibitory effects on HepG2 cell migration and invasion. After treating with ATRA or ATRA-NPs, dose-dependent inhibitions on HepG2 cell migration and invasion were detected (Fig. 3f and j). Compared with ATRA, ATRA-NPs generated significant inhibitory effects on HepG2 cell migration and invasion (Fig. 3j). The results demonstrated that ATRA-NPs could significantly enhance the inhibitory effect of ATRA on cancer migration and invasion.

Further, the inhibition of ATRA-NPs on cancer cell motility was investigated by wound healing assay. As shown in Fig. 3g and k, the wound healing rate of HuH7 cells in the control group (untreated cells) was 91 ± 3%, indicating that HuH7 cells possessed strong migration ability. After treating with ATRA or ATRA-NPs, the wound healing rate of HuH7 cells decreased in a dose-dependent manner (Fig. 3g and k). In comparison, the inhibitory effect of ATRA-NPs on wound healing was significantly higher than that of ATRA (Fig. 3k). Similar inhibitory behaviors of the ATRA, and ATRA-NPs on wound healing were detected in HepG2 cells. As shown in Fig. 3h and l, the wound healing rate of HepG2 cells in the control group (untreated cells) was 95 ± 2%, indicating that HepG2 cells also presented strong migration ability. After treating with ATRA or ATRA-NPs, the wound healing of HepG2 cells was inhibited in a dose-dependent manner (Fig. 3h and l). In comparison, the inhibitory effect of ATRA-NPs on wound healing in HepG2 cells was significantly higher than that of ATRA (Fig. 3l). Overall, these results demonstrated that ATRA-NPs could significantly enhance the inhibition of ATRA on cancer metastasis, and the nanoparticle itself displayed excellent biocompatibility.

### Inhibition of ATRA-NPs on cancer cell Pin1 and signaling pathways

**Figure 4.**
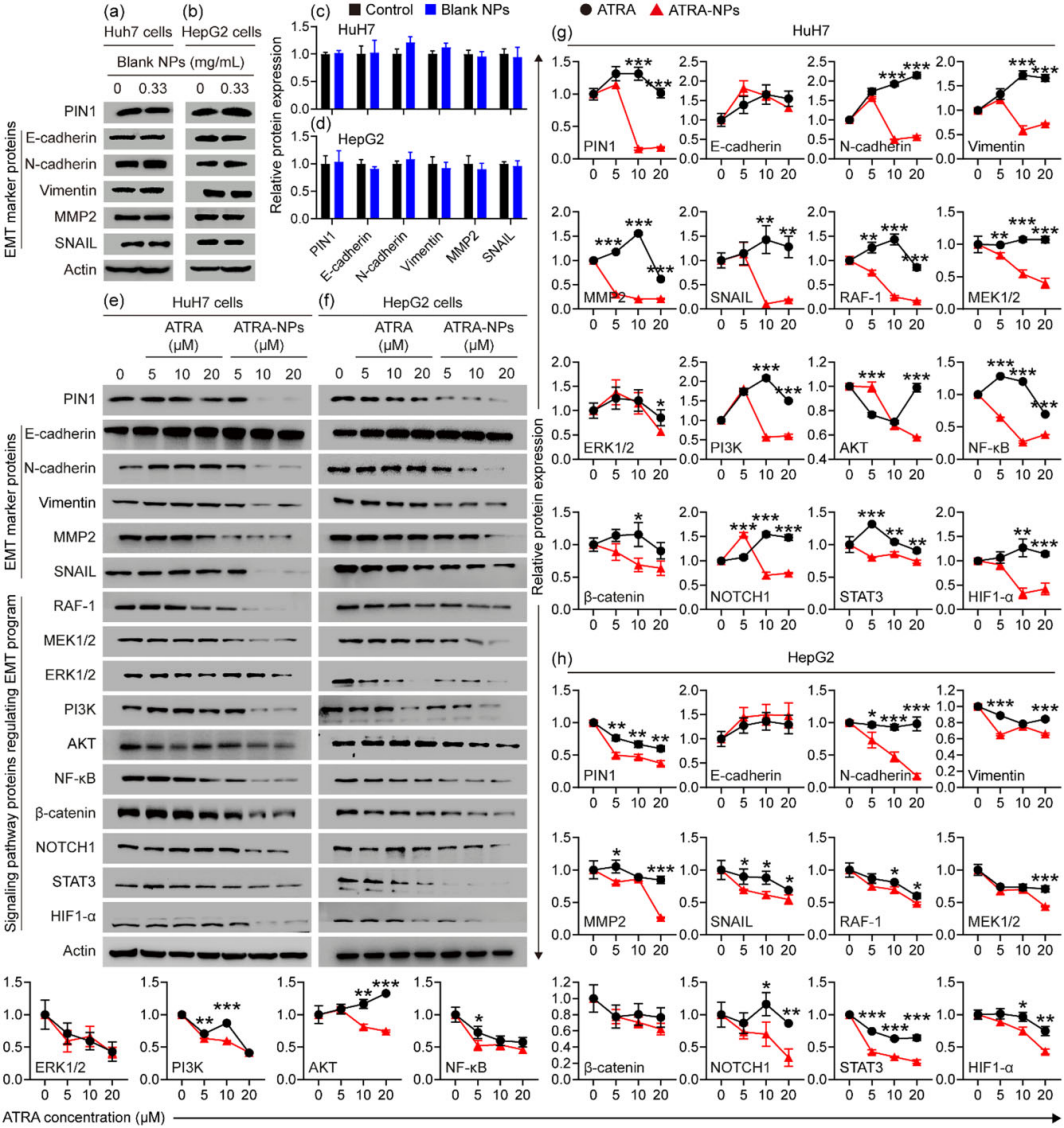
Effects of nanoparticles on PIN1 and signaling pathway proteins in cancer cells. (**a**) Western blot images and (**c**) relative expression values of PIN1 and EMT marker proteins in HuH7 cells after treatment with 0.33 mg ml^−1^ blank NPs for 72 h (*n* = 3). (**b**) Western blot images and (**d**) relative expression values of PIN1 and EMT marker proteins in HepG2 cells after treatment with 0.33 mg ml^−1^ blank NPs for 72 h (*n* = 3). (**e**) Western blot images and (**g**) relative expression values of PIN1, EMT marker proteins and signaling pathway proteins in HuH7 cells after 72 h of treatment (*n* = 3). (**f**) Western blot images and (**h**) relative expression values of PIN1, EMT marker proteins and signaling pathway proteins in HepG2 cells after 72 h of treatment (*n* = 3).

To understand the mechanism of ATRA-NPs against tumor metastasis, the effect of the nanoparticles on the expression of Pin1, EMT marker proteins and signaling pathway proteins in cancer cells was investigated. As depicted in Fig. 4a and c, the blank NPs had no inhibitory effect on the Pin1 and EMT marker proteins of HuH7 cells compared with the control group. Figure 4e and g show the relative expression levels of Pin1, EMT marker proteins and signaling pathway proteins in HuH7 cells after treatment with ATRA or ATRA-NPs. Compared with the control group, ATRA had almost no inhibitory effect on Pin1, EMT marker proteins and signaling pathway proteins, except that ATRA produced some inhibition on MMP2 and NF-kB at a concentration of 20 µM. In contrast, ATRA-NPs produced dose-dependent inhibitory effects on Pin1, EMT marker proteins (N-cadherin, Vimentin, MMP2 and SNAIL) and signaling pathway proteins (RAF-1, MEK, ERK, PI3K, AKT, NF-kB, β-catenin, NOTCH1, STAT3 and HIF1-α). In comparison, the inhibitory effects of ATRA-NPs on Pin1, EMT marker proteins (N-cadherin, Vimentin, MMP2 and SNAIL) and signaling pathway proteins (RAF-1, MEK, ERK, PI3K, AKT, NF-kB, β-catenin, NOTCH1, STAT3 and HIF1-α) were significantly higher than that of ATRA. In addition, the treatments were able to significantly upregulate HuH7 cell E-cadherin. Similar effects of the blank NPs, ATRA and ATRA-NPs on Pin1, EMT marker proteins and signaling pathway proteins were detected in HepG2 cells. As shown in Fig. 4b and d, the blank NPs had almost no inhibitory effect on Pin1 and EMT marker proteins in HepG2 cells. After treatment with ATRA-NPs, Pin1, EMT marker proteins (N-cadherin, Vimentin, MMP2 and SNAIL) and signaling pathway proteins (RAF-1, MEK, ERK, PI3K, AKT, NF-kB, β-catenin, NOTCH1, STAT3 and HIF1-α) of HepG2 cells were downregulated in a dose-dependent manner (Fig. 4f and h). In comparison, the inhibitory effects of ATRA-NPs on Pin1, EMT marker proteins (N-cadherin, Vimentin, MMP2 and SNAIL) and signaling pathway proteins (RAF-1, MEK, PI3K, AKT, NF-kB, NOTCH1, STAT3 and HIF1-α) were significantly higher than that of ATRA (Fig. 4h). Overall, these results demonstrated that ATRA-NPs could significantly enhance the inhibitory effect of ATRA on Pin1, EMT marker proteins (N-cadherin, Vimentin, MMP2 and SNAIL) and signaling pathway proteins (RAF-1, MEK, ERK, PI3K, AKT, NF-kB, β-catenin, NOTCH1, STAT3 and HIF1-α) in cancer cells. At the same time, the nanoparticles themselves showed no effect on Pin1 and EMT marker proteins. In addition, ATRA-NPs could upregulate E-cadherin in cancer cells.

Since Pin1 can activate >50 oncogenes [22, 23], inhibition of Pin1 will result in the inhibition of its substrate oncogenes. Indeed, oncogenes activated by Pin1, including RAF-1, AKT, NF-kB, β-catenin, NOTCH1, STAT3 and HIF1-α, were downregulated in ATRA-NPs-treated cancer cells in a dose-dependent manner. RAF proteins are key components of the RAF/MEK/ERK signaling pathway. This pathway is commonly dysregulated in several human cancers and plays an important role in cancer metastasis [41–43]. The PI3K/AKT signaling pathway is abnormally activated in a variety of cancers and controls cancer hallmarks such as cancer cell growth, metastasis, EMT and drug resistance [44, 45]. In addition, the PI3K/AKT signaling pathway cross-talks with other signaling pathways such as NF-kB signaling pathway [44, 46]. The WNT/β-catenin signaling pathway is commonly overactivated in cancer, which promotes cancer growth and dissemination [47, 48]. The NOTCH signaling pathway plays a vital role in cancer initiation and promotes cancer metastasis by enhancing EMT progression [49, 50]. STAT3 is overexpressed and constitutively activated in various cancers, which promotes cancer initiation and progression [51, 52]. HIF1α is widely expressed in many human cancers, and activation of HIF1α signaling contributes to cancer cell survival and metastasis [53, 54]. Furthermore, the RAF/MEK/ERK, PI3K/AKT/NF-kB, WNT/β-catenin, NOTCH, STAT3 and HIF1α signaling pathways play critical regulatory roles in EMT progression, and these signaling pathways can induce full EMT responses through cooperation [6]. The downregulation of RAF-1, MEK, ERK, PI3K, AKT, NF-kB, β-catenin, NOTCH1, STAT3 and HIF1-α will lead to inhibition of these signaling pathways, which will block cancer EMT progression. The downregulation of E-cadherin and the upregulation of N-cadherin are hallmarks of EMT [6]. Moreover, increased expressions of Vimentin, matrix metalloproteinase MMP2 and transcription factor SNAIL are essential for the initiation and progression of EMT [6]. Conversely, the upregulation of E-cadherin and the downregulation of N-cadherin, Vimentin, MMP2 and SNAIL would indicate the inhibited EMT progression. As expected, ATRA-NPs treatment significantly downregulated N-cadherin, Vimentin, MMP2 and SNAIL and upregulated E-cadherin. These outcomes demonstrated that ATRA-NPs treatment could inhibit cancer EMT progression. Collectively, these results consistently suggested that ATRA-NPs inhibited the cancer metastasis via downregulating Pin1 and then simultaneously blocking signaling pathways and cancer EMT progression.

### Effect of Pin1 knockdown on anti-metastatic efficacy of ATRA-NPs

**Figure 5.**
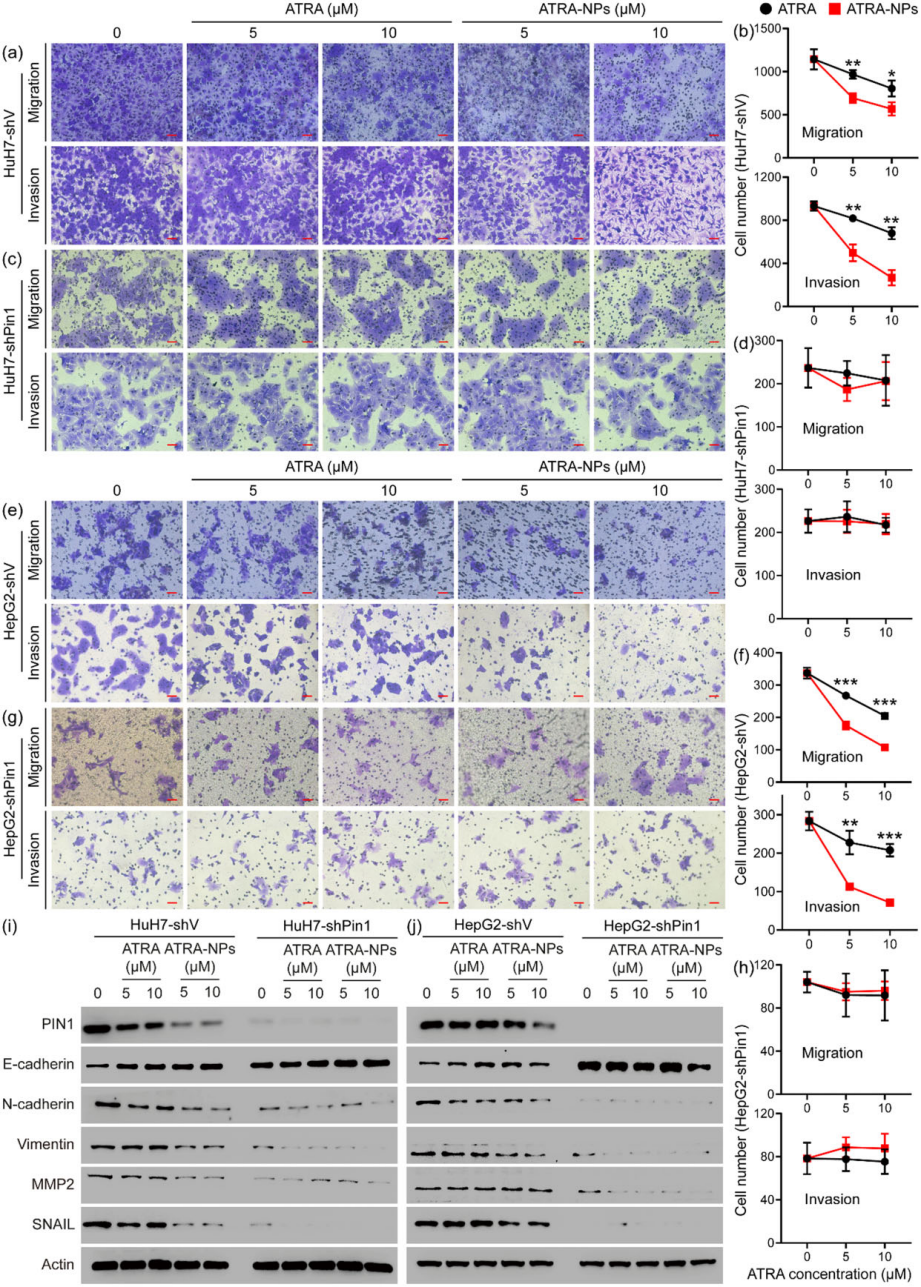
Effect of Pin1 knockdown on anti-metastatic efficacy of ATRA-NPs. (**a**) Images and (**b**) numbers of migrated and invaded HuH7-shV cells after 72 h of treatment (*n* = 3). (**c**) Images and (**d**) numbers of migrated and invaded HuH7-shPin1 cells after 72 h of treatment (*n* = 3). (**e**) Images and (**f**) numbers of migrated and invaded HepG2-shV cells after 72 h of treatment (*n* = 3). (**g**) Images and (**h**) numbers of migrated and invaded HepG2-shPin1 cells after 72 h of treatment (*n* = 3). (**i**) Western blot images of PIN1 and EMT marker proteins from HuH7-shV and HuH7-shPin1 cells after 72 h of treatment (*n* = 3). (**j**) Western blot images of PIN1 and EMT marker proteins from HepG2-shV and HepG2-shPin1 cells after 72 h of treatment (*n* = 3).

To confirm whether Pin1 is the main target for ATRA-NPs, we investigated the effect of Pin1 knockdown on the anti-metastatic efficacy of ATRA-NPs. As shown in Supplementary Fig. S5, migration and invasion of HuH7 cells expressing empty vector (HuH7-shV) were not inhibited compared to wild-type cells. In contrast, Pin1 knockdown (shPin1) significantly reduced the migration and invasion of HuH7 cells. Figure 5a–d shows the migration and invasion of HuH7-shV and HuH7-shPin1 after ATRA or ATRA-NPs treatment. For HuH7-shV cells, ATRA or ATRA-NPs treatment produced a dose-dependent inhibitory effect on cell migration and invasion. In comparison, ATRA-NPs had a significantly higher inhibitory effect on the migration and invasion of HuH7-shV cells than ATRA (Fig. 5b). However, for HuH7-shPin1 cells, neither ATRA-NPs nor ATRA treatment had a significant inhibitory effect on cell migration and invasion (Fig. 5c and d). This suggests that Pin1 knockdown almost eliminates the inhibitory effect of ATRA-NPs on HuH7 cell migration and invasion. Figure 5i shows the levels of PIN1 and EMT marker proteins in HuH7-shV and HuH7-shPin1 cells after ATRA or ATRA-NPs treatment. For HuH7-shV cells, ATRA treatment slightly downregulated PIN1, N-cadherin and SNAIL, but upregulated E-cadherin. In contrast, ATRA-NPs treatment greatly downregulated PIN1, N-cadherin, Vimentin, MMP2 and SNAIL, and further upregulated E-cadherin. These outcomes indicated that ATRA-NPs could enhance the inhibitory effect of ATRA on PIN1 and EMT progression in cancer cells, which was consistent with the above experimental results. Compared with HuH7-shV cells, Pin1 knockdown greatly upregulated E-cadherin but significantly downregulated N-cadherin, Vimentin, MMP2 and SNAIL. Moreover, neither ATRA nor ATRA-NPs treatment affected the levels of EMT marker proteins in HuH7-shPin1 cells (Fig. 5i), indicating that Pin1 was the main target of ATRA-NPs against cancer metastasis.

Similar effects of ATRA and ATRA-NPs on cell migration and invasion were detected in HepG2-shV and HepG2-shPin1 cells. As depicted in Fig. 5e and f, ATRA or ATRA-NPs treatment inhibited the migration and invasion of HepG2-shV cells in a dose-dependent manner, and the inhibitory effect by ATRA-NPs was significantly higher than that by ATRA. However, for HepG2-shPin1 cells, neither ATRA nor ATRA-NPs treatment showed no inhibitory effect on cell migration and invasion (Fig. 5g and h). ATRA-NPs treatment significantly reduced PIN1, N-cadherin, Vimentin, MMP2 and SNAIL in HepG2-shV cells but increased E-cadherin (Fig. 5j). However, neither ATRA nor ATRA-NPs treatment affected the levels of EMT marker proteins in HepG2-shPin1 cells. These results demonstrated that Pin1 knockdown eliminated the anti-metastatic efficacy of ATRA-NPs. Overall, these results confirmed that Pin1 played a key role in cancer metastasis and was the main target of ATRA-NPs against cancer metastasis.

### *In vivo* anti-tumor metastasis, pharmacokinetics and biodistribution

**Figure 6.**
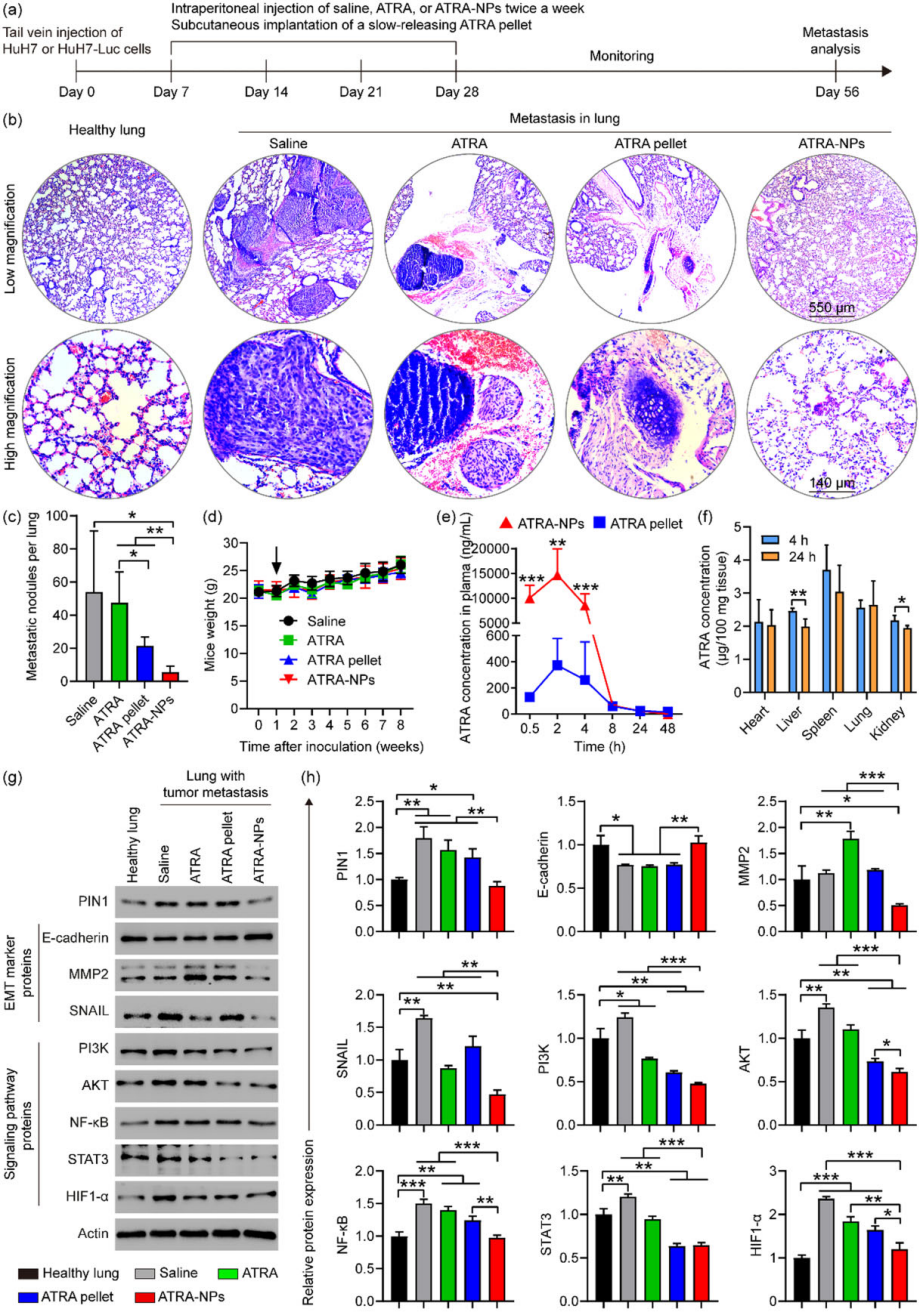
*In vivo* anti-tumor metastasis, pharmacokinetics and biodistribution. (**a**) Illustration of cancer cell inoculation, drug treatment and evaluation of tumor metastasis therapy in mice. (**b**) H&E staining photos of lung tissue sections and (**c**) metastatic nodules on each lung of mice after different treatments (*n* = 4 ∼ 5). (**d**) Body weight of mice in each group (*n* = 5). (**e**) The concentration of ATRA in the plasma of mice after different treatments (*n* = 4). (**f**) Biodistribution of ATRA-NPs in mice after 4 and 24 h of administration (*n* = 4). (**g**) Western blot images and (**h**) relative expression values of PIN1, EMT marker proteins and signaling pathway proteins in lung tissue of mice after different treatments (*n* = 3).

To further determine the anti-metastatic efficacy and mechanism of ATRA-NPs, we investigated the inhibition of ATRA-NPs on lung metastasis of cancer cells in mice. The construction, drug treatment and therapeutic evaluation of a mouse model with lung metastases were carried out (Fig. 6a). To monitor the distribution of tumor cells in mice, HuH7-Luc cells were established (Supplementary Fig. S6a). The results of bioluminescence imaging showed that HuH7 tumor cells were mainly distributed in the lung tissue of mice (Supplementary Fig. S6b). Supplementary Figs S7 and 6b and c show the lung photos, H&E staining photos of lung tissue sections, and metastatic nodules on each lung of mice after different treatments. Numerous metastatic nodules were observed on lungs from the saline group compared to healthy lungs, which confirmed that mouse models with lung metastases were successfully established. Compared with the saline group, ATRA and ATRA pellet exerted a slight and moderate inhibitory effect on lung metastasis, respectively. The number of metastatic nodules on each lung from the ATRA-NPs group was 6 ± 4. In contrast, the saline, ATRA and ATRA pellet groups resulted in 54 ± 37, 47 ± 19 and 22 ± 5, respectively (Fig. 6c). Moreover, the size of metastatic nodules in ATRA-NPs group was much smaller than that in saline, ATRA and ATRA pellet groups (Fig. 6b). These results indicated that ATRA-NPs could significantly enhance the inhibition of ATRA on lung metastasis of cancer cells. Compared with the saline group, treatment with ATRA or ATRA pellet or ATRA-NPs showed no signs of a significant reduction in the body weight of mice (Fig. 6d), indicating good biocompatibility of both ATRA and ATRA-NPs.

To further understand why ATRA-NPs enhance the anti-metastatic efficacy of ATRA, we measured the pharmacokinetics of ATRA-NPs and compared them with commercially available ATRA sustained-release pellets. As shown in Fig. 6e, the concentration of ATRA in plasma of ATRA-NPs group was significantly higher than that of ATRA pellet group. Moreover, the maximal plasma concentration (*C_max_*) and area under the plasma concentration–time curve of ATRA-NPs group were significantly higher than those of ATRA pellet group (Supplementary Table S2). These results demonstrated that the pharmacokinetic performance of ATRA-NPs was significantly higher than that of ATRA sustained-release pellets. Moreover, we further determined the biodistribution of ATRA-NPs in mice. As shown in Fig. 6f, ATRA-NPs accumulated mainly in the spleen and lung tissues after 4 and 24 h of administration. Compared with the 4 h group, the content of ATRA-NPs in the lung, heart and spleen did not decrease significantly in the 24 h group except for a small decrease in the content of ATRA-NPs in the liver and kidney. This result further confirmed that ATRA-NPs can release ATRA slowly *in vivo*. Overall, ATRA-NPs could slowly release ATRA and be internalized by cancer cells, which explains the anti-metastatic efficacy of ATRA-NPs.

**Figure 7.**
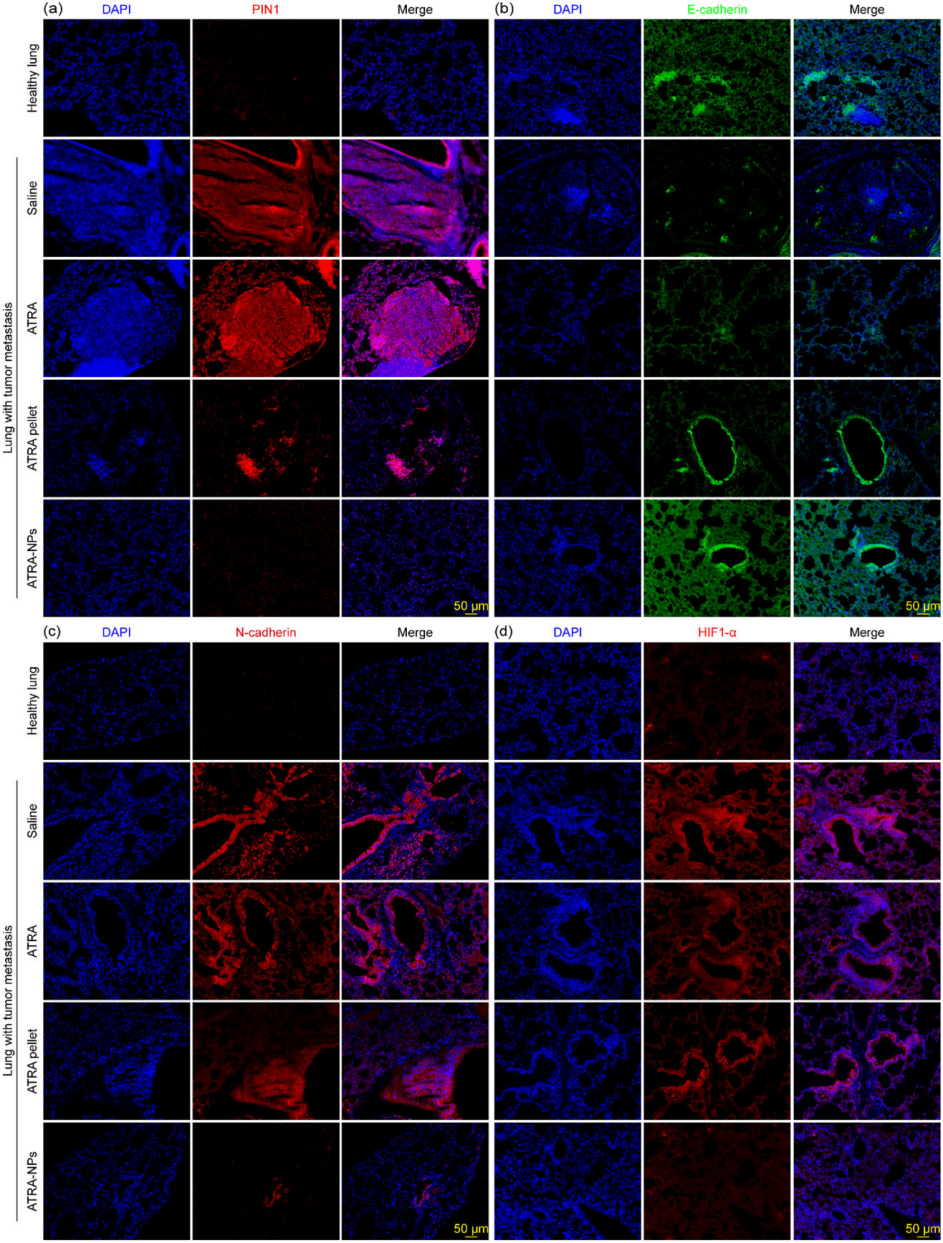
Immunofluorescence images of (**a**) PIN1, (**b**) E-cadherin, (**c**) N-cadherin and (**d**) HIF1-α in lung tissue after different treatments.

To further determine the mechanism of anti-tumor metastasis of ATRA-NPs, we applied Western blot and immunohistofluorescence analyses to investigate the expression of PIN1, EMT marker proteins and signaling pathway proteins in lung tissue of mice after different treatments. As shown in Fig. 6g and h, compared with healthy lungs, PIN1, SNAIL, PI3K, AKT, NF-kB, STAT3 and HIF1-α in lungs from the saline group were significantly upregulated, while E-cadherin was significantly downregulated. These results indicated that the EMT program and signaling pathways in the lungs of the saline group were activated. Compared with the saline group, the treatment of ATRA and ATRA pellets had slight and moderate inhibition on PIN1, PI3K, AKT, NF-kB, STAT3 and HIF1-α in the lung, respectively, but had no inhibition on E-cadherin and MMP2. In contrast, ATRA-NPs treatment significantly downregulated PIN1, MMP2, SNAIL, PI3K, AKT, NF-kB, STAT3 and HIF1-α, and very significantly upregulated E-cadherin. Moreover, the inhibition of ATRA-NPs on PIN1, MMP2, SNAIL, PI3K, AKT, NF-kB and HIF1-α was significantly higher than that of ATRA or ATRA pellet. In addition, levels of PIN1, E-cadherin, NF-kB and HIF1-α in the ATRA-NPs group were comparable to those of healthy lungs. The effects of ATRA, ATRA pellet and ATRA-NPs on PIN1, EMT marker proteins and signaling pathway proteins in lungs with tumor metastasis were further confirmed by immunohistofluorescence analysis. As shown in Fig. 7 and Supplementary Figs. S8–10, PIN1, N-cadherin, Vimentin, MMP2, SNAIL and HIF1-α were significantly upregulated and E-cadherin was considerably downregulated in the saline group compared to healthy lungs. Compared with the saline group, ATRA treatment had no significant effect on PIN1, E-cadherin, N-cadherin, Vimentin, MMP2, SNAIL and HIF1-α, while ATRA pellet treatment produced slight or moderate inhibition on PIN1, N-cadherin, Vimentin, MMP2, SNAIL and HIF1-α. In contrast, ATRA-NPs treatment greatly downregulated PIN1, N-cadherin, Vimentin, MMP2, SNAIL and HIF1-α, and significantly upregulated E-cadherin. In addition, levels of PIN1, E-cadherin, N-cadherin, Vimentin, MMP2, SNAIL and HIF1-α in the ATRA-NPs group were comparable to those of healthy lungs. Overall, these results consistently demonstrated that ATRA-NPs significantly enhance the inhibitory effect of ATRA on Pin1 expression, EMT progression and signaling pathways in lungs with tumor metastasis, which was consistent with anti-metastasis results *in vitro*.

### *In vivo* biosafety of ATRA-NPs

To further determine the *in vivo* biosafety of ATRA-NPs, blood biochemical index analysis and H&E staining of major organs of mice were performed after different treatments. As depicted in Supplementary Fig. S11, compared with normal mice, the levels of creatine kinase (CK), lactate dehydrogenase (LDH), alanine aminotransferase (ALT), aspartate aminotransferase (AST), total bilirubin (TBIL), serum albumin (ALB) and creatinine (CREA) in mice with lung metastases showed no signs of changes. However, the level of blood urea nitrogen (BUN) was significantly downregulated. Compared with the saline group, ATRA or ATRA pellet or ATRA-NPs treatment had no significant effect on CK, LDH, ALT, AST, TBIL, ALB, BUN and CREA in mice. Considering CK and LDH as the functional indices of the heart, and ALT, AST, TBIL, ALB, BUN and CREA related to liver and kidney functions, these results suggested that lung metastasis of cancer cells might have some effect on liver and kidney functions in mice. However, the treatment of ATRA or ATRA pellet or ATRA-NPs showed no effects on the heart, liver and kidney functionalities of mice. The H&E staining results showed that, compared with normal mice, no noticeable histological abnormalities or damages were observed in the heart, liver, spleen and kidney of mice after different treatments (Supplementary Fig. S12). These results further indicated that the treatment with ATRA or ATRA pellet or ATRA-NPs did not cause apparent damage to the major organs of mice. Together, these results demonstrated the excellent biosafety of ATRA-NPs *in vivo*.

## Conclusion

**Figure 8.**
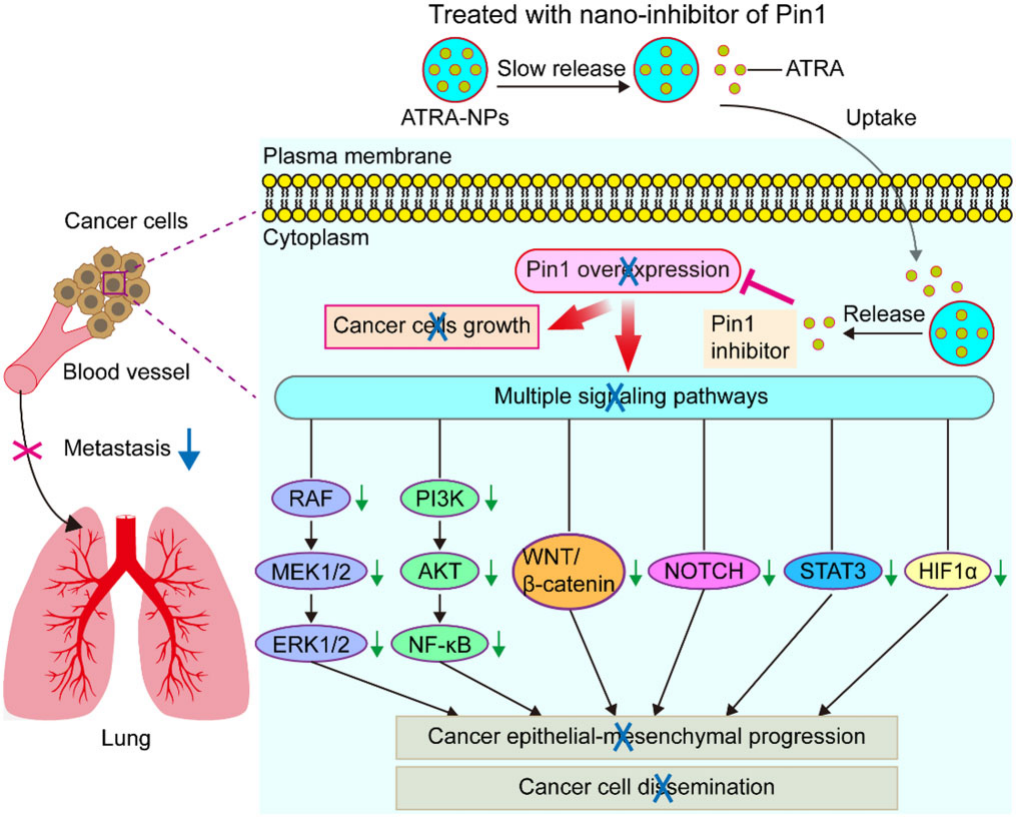
Schematic illustrating the mechanistic views of anti-tumor metastasis of ATRA-NPs.

In summary, a new nano-drug of Pin1 inhibitor was developed through a one-step method using supercritical CO_2_ processing. The optimal experimental conditions for preparing ATRA-NPs with good spherical morphology, smooth surface, minimum particle size, as well as maximum DL and EE were determined as follows: ratio of ATRA and PLA–PEG–PLA = 3%, PLA–PEG–PLA concentration = 0.5%, ratio of DCM and acetone = 0.5, and flow rate of solution = 0.5 ml min^−1^. ATRA-NPs could effectively and slowly release ATRA. In addition, ATRA-NPs showed excellent internalization by cancer cells. ATRA-NPs exhibited excellent biosafety, and could not only significantly enhance the inhibition of ATRA on cancer growth and reduce its dose, but also significantly augment the inhibition of ATRA on cancer metastasis. Pin1 played a key role in cancer metastasis and was the main target of ATRA-NPs against cancer metastasis. ATRA-NPs exerted their potent anti-metastatic effect by inhibiting Pin1 and then simultaneously blocking multiple signaling pathways and cancer epithelial–mesenchymal progression (Fig. 8). Since ATRA-NPs could effectively couple the inhibition of cancer cell dissemination with the inhibition of cancer growth, it provided a novel therapeutic strategy for efficiently inhibiting cancer metastasis.

## Supplementary Material

rbad014_Supplementary_Data
