# Correction to: Nanocarrier of Pin1 inhibitor based on supercritical fluid technology inhibits cancer metastasis by blocking multiple signaling pathways

**DOI:** 10.1093/rb/rbae132

**Published:** 2024-11-19

This is a correction to: Fengzhu Zhang, Aiwen Zhang, Youning Xie, Haiying Wen, Ranjith Kumar Kankala, Jing Huang, Anjun Zhang, Qi Wang, Biaoqi Chen, Haiyan Dong, Zhao Guo, Aizheng Chen, Dayun Yang, Nanocarrier of Pin1 inhibitor based on supercritical fluid technology inhibits cancer metastasis by blocking multiple signaling pathways, *Regenerative Biomaterials*, Volume 10, 2023, rbad014, https://doi.org/10.1093/rb/rbad014

In the originally published version of this article, Figure 3 contained the following duplication errors, inadvertently introduced during figure assembly:

1. The image labeled “Figure 3g - HuH7 cell - 0 h - ATRA (5 µM)” was incorrect. It was mistakenly replaced with the image for “Figure 3g - HuH7 cell - 0 h - ATRA-NPs (10 µM).”
2. The image labeled “Figure 3f - HepG2 cell - Invasion - 0” was incorrect. It was mistakenly replaced with the image for “Figure 3c - HepG2 cell - Invasion - Control.”

These errors have now been corrected.

