# Supplementary material for: Nanocarrier of Pin1 inhibitor based on supercritical fluid technology inhibits cancer metastasis by blocking multiple signaling pathways
Source: Regen Biomater. 2023 Feb 27;10:rbad014. doi: 10.1093/rb/rbad014 (PMC10008082; doi:10.1093/rb/rbad014)
Supplement: rbad014_Supplementary_Data [file rbad014_supplementary_data.docx]

**Supplementary Tables and Figures**

**Nanocarrier of Pin1 inhibitor based on supercritical fluid technology inhibits cancer metastasis by blocking multiple signaling pathways**

Fengzhu Zhang ^a1^, Aiwen Zhang ^a1^, Youning Xie ^a1^, Haiying Wen ^a^, Ranjith Kumar Kankala ^b,c^, Jing Huang ^a^, Anjun Zhang ^a^, Qi Wang ^a^, Biaoqi Chen ^b,c^, Haiyan Dong ^a^, Zhao Guo ^a^, Aizheng Chen ^b,c^, Dayun Yang ^a^^[[1]](#footnote-1)^

*^a^ Fujian Key Laboratory of Translational Research in Cancer and Neurodegenerative Diseases, School of Basic Medical Sciences, Fujian Medical University, Fuzhou, 350108, PR China*

*^b^ Institute of Biomaterials and Tissue Engineering, Huaqiao University, Xiamen, 361021, PR China*

*^c^ Fujian Provincial Key Laboratory of Biochemical Technology (Huaqiao University), Xiamen, 361021, PR China*

**Table S1**

Experimental results from the factorial design (-1 and 1 represent low and high factor settings in the experimental design, respectively).

| Run order | Factor A | Factor B | Factor C | Factor D | Particle size (nm) | DL (%) | EE (%) |
| --- | --- | --- | --- | --- | --- | --- | --- |
| 1 | -1 | 1 | -1 | -1 | 907±484 | 0.5±0.0 | 77.6±1.3 |
| 2 | 1 | -1 | 1 | 1 | 1524±713 | 0.6±0.0 | 96.1±0.3 |
| 3 | 1 | -1 | -1 | -1 | 633±526 | 1.8±0.0 | 96.8±0.2 |
| 4 | 1 | 1 | 1 | 1 | 1210±648 | 1.7±0.0 | 99.6±0.1 |
| 5 | 1 | 1 | -1 | 1 | 1063±536 | 1.3±0.0 | 98.4±0.1 |
| 6 | -1 | 1 | 1 | 1 | 997±522 | 0.3±0.0 | 100.0±0.0 |
| 7 | 1 | -1 | -1 | 1 | 725±536 | 0.7±0.0 | 90.8±0.4 |
| 8 | -1 | 1 | -1 | 1 | 993±536 | 0.2±0.0 | 98.7±0.5 |
| 9 | 1 | -1 | 1 | -1 | 728±355 | 1.1±0.0 | 98.4±0.1 |
| 10 | -1 | -1 | -1 | 1 | 372±129 | 0.3±0.0 | 83.2±0.5 |
| 11 | 1 | 1 | -1 | -1 | 632±269 | 1.8±0.0 | 93.9±0.5 |
| 12 | -1 | -1 | 1 | -1 | 719±356 | 0.3±0.1 | 100.0±0.0 |
| 13 | -1 | -1 | 1 | 1 | 1114±564 | 0.1±0.0 | 100.0±0.0 |
| 14 | -1 | 1 | 1 | -1 | 1182±834 | 0.4±0.0 | 88.9±1.1 |
| 15 | -1 | -1 | -1 | -1 | 446±216 | 0.1±0.0 | 88.8±2.9 |
| 16 | 1 | 1 | 1 | -1 | 1066±741 | 2.0±0.0 | 96.2±0.1 |

**Table S2**

Pharmacokinetic parameters of ATRA in BALB/c nu/nu mice after implantation of ATRA pellet or injection of ATRA-NPs (mean ± SD, n = 4).

| Parameter | ATRA pellet | ATRA-NPs |
| --- | --- | --- |
| *C*_max_ (μg/L) | 405.46 ± 224.004 | 14717.658 ± 5215.899** |
| *AUC*_(0−_*_t_*_)_ (μg/L×h) | 2736.043 ± 1223.076 | 104422.727 ± 65921.788* |
| *t*_1/2_ (h) | 15.557 ± 4.866 | 2.347 ± 0.033** |

* *p* ˂ 0.05 between the ATRA-NPs and ATRA pellet.

** *p* ˂ 0.01 between the ATRA-NPs and ATRA pellet.


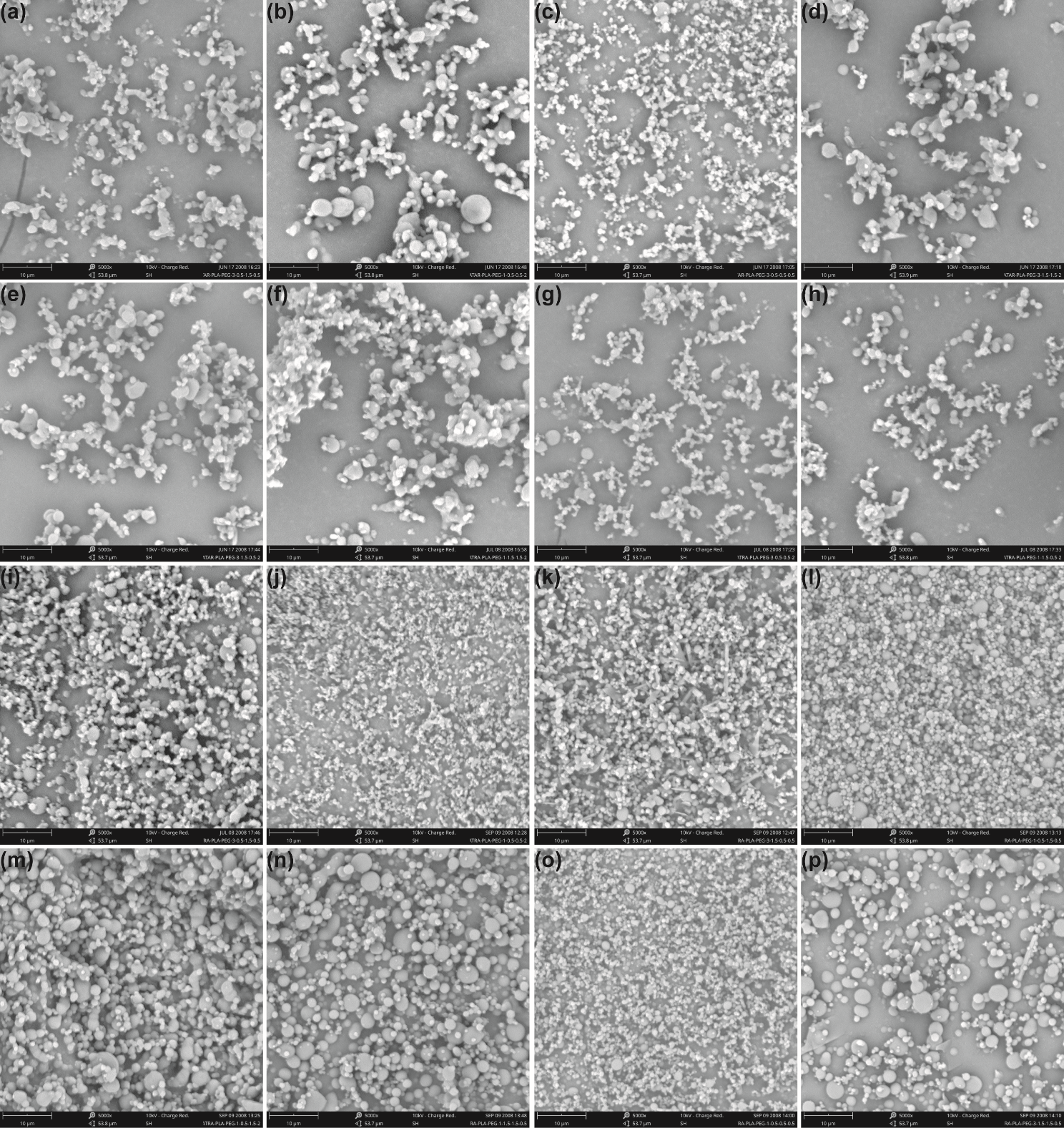


Figure S1. SEM images of ATRA-NPs prepared by the SAS process under different conditions (a) 1%, 1.5%, 0.5, and 0.5 mL/min; (b) 3%, 0.5%, 1.5, and 2 mL/min; (c) 3%, 0.5%, 0.5, and 0.5 mL/min; (d) 3%, 1.5%, 1.5, and 2 mL/min; (e) 3%, 1.5%, 0.5, and 2 mL/min; (f) 1%, 1.5%, 1.5, and 2 mL/min; (g) 3%, 0.5%, 0.5, and 2 mL/min; (h) 1%, 1.5%, 0.5, and 2 mL/min; (i) 3%, 0.5%, 1.5, and 0.5 mL/min; (j) 1%, 0.5%, 0.5, and 2 mL/min; (k) 3%, 1.5%, 0.5, and 0.5 mL/min; (l) 1%, 0.5%, 1.5, and 0.5 mL/min; (m) 1%, 0.5%, 1.5, and 2 mL/min; (n) 1%, 1.5%, 1.5, and 0.5 mL/min; (o) 1%, 0.5%, 0.5, and 0.5 mL/min; (p) 3%, 1.5%, 1.5, and 0.5 mL/min (the parameters are ratio of ATRA and PLA-PEG-PLA (%), PLA-PEG-PLA concentration (%), ratio of dichloromethane and acetone (v/v), and flow rate of solution (mL/min), respectively).


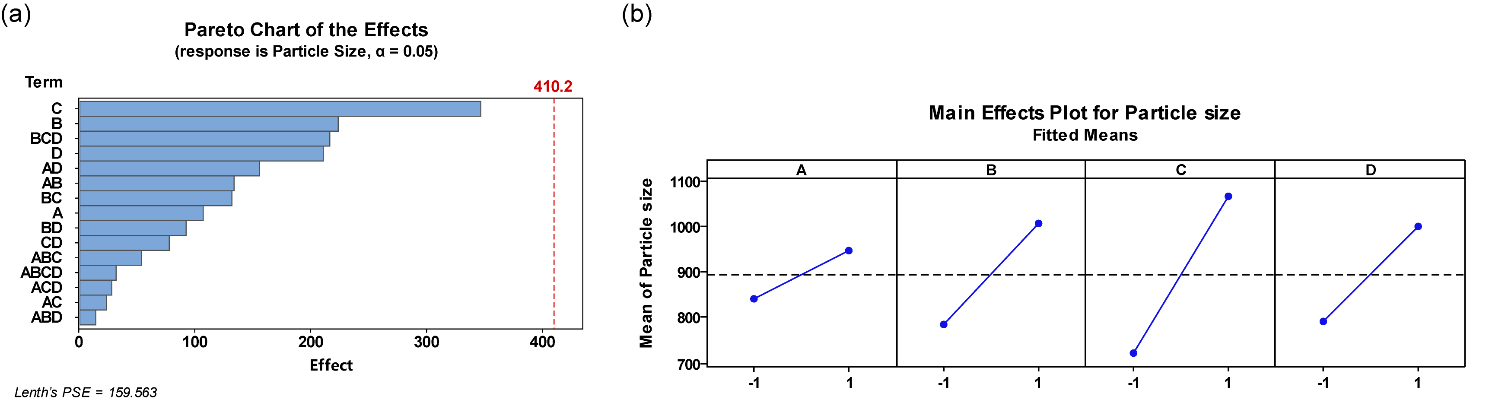


Figure S2. (a) Effect of the factors on particle size. Factors A, B, C and D represent the ratio of ATRA and PLA-PEG-PLA (%), PLA-PEG-PLA concentration (%), ratio of dichloromethane and acetone (v/v), and flow rate of solution (mL/min) in the SAS process, respectively. (b) Main effects plot for particle size. The -1 and 1 value of the X axis respectively represent the low and high factor settings in the experimental design.


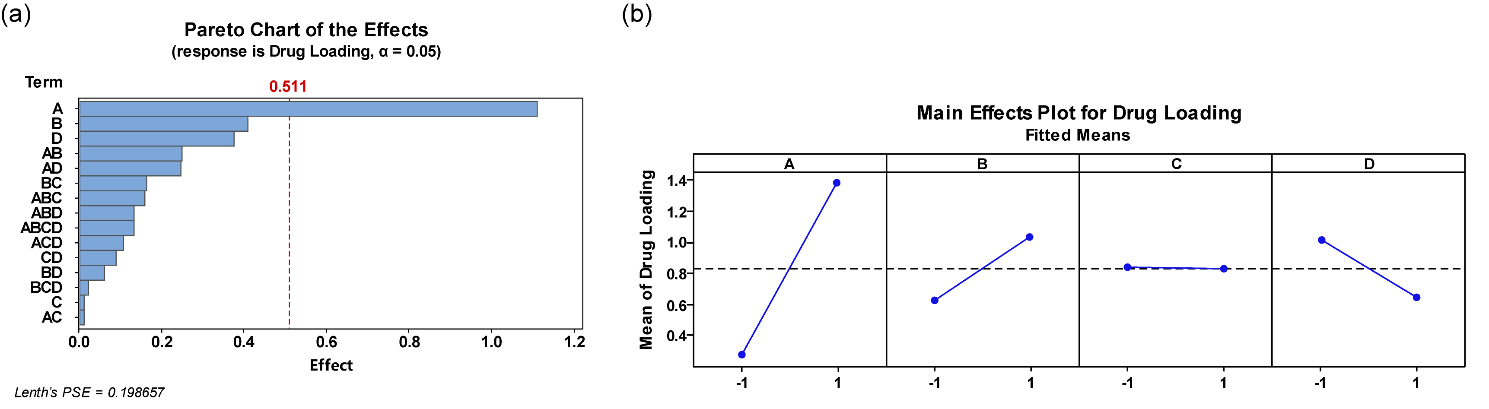


Figure S3. (a) Effect of the factors on drug loading (DL). Factors A, B, C and D represent the ratio of ATRA and PLA-PEG-PLA (%), PLA-PEG-PLA concentration (%), ratio of dichloromethane and acetone (v/v), and flow rate of solution (mL/min) in the SAS process, respectively. (b) Main effects plot for DL. The -1 and 1 value of the X axis respectively represent the low and high factor settings in the experimental design.


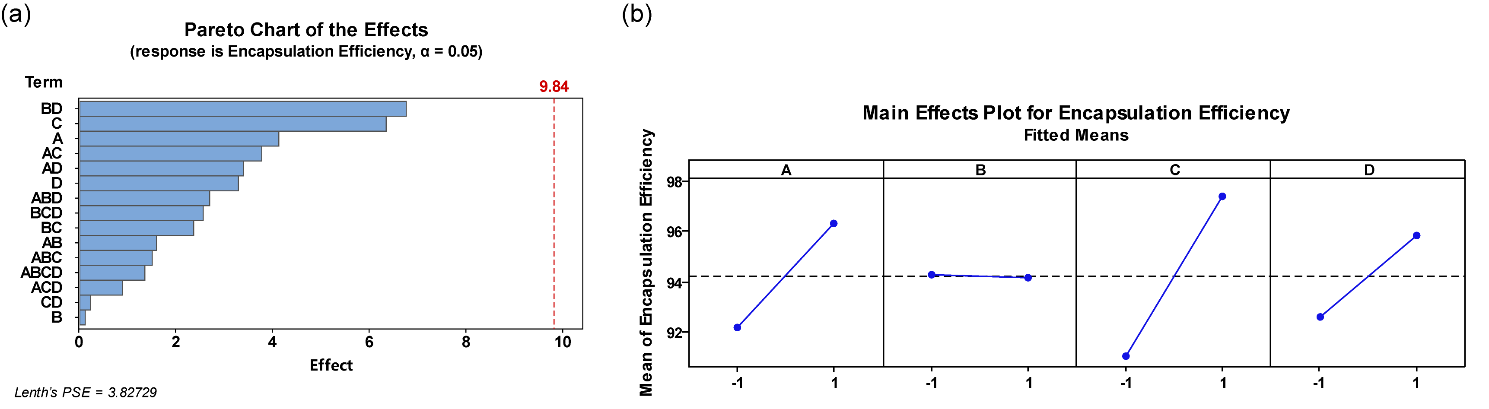


Figure S4. (a) Effect of the factors on encapsulation efficiency (EE). Factors A, B, C and D represent the ratio of ATRA and PLA-PEG-PLA (%), PLA-PEG-PLA concentration (%), ratio of dichloromethane and acetone (v/v), and flow rate of solution (mL/min) in the SAS process, respectively. (b) Main effects plot for EE. The -1 and 1 value of the X axis respectively represent the low and high factor settings in the experimental design.


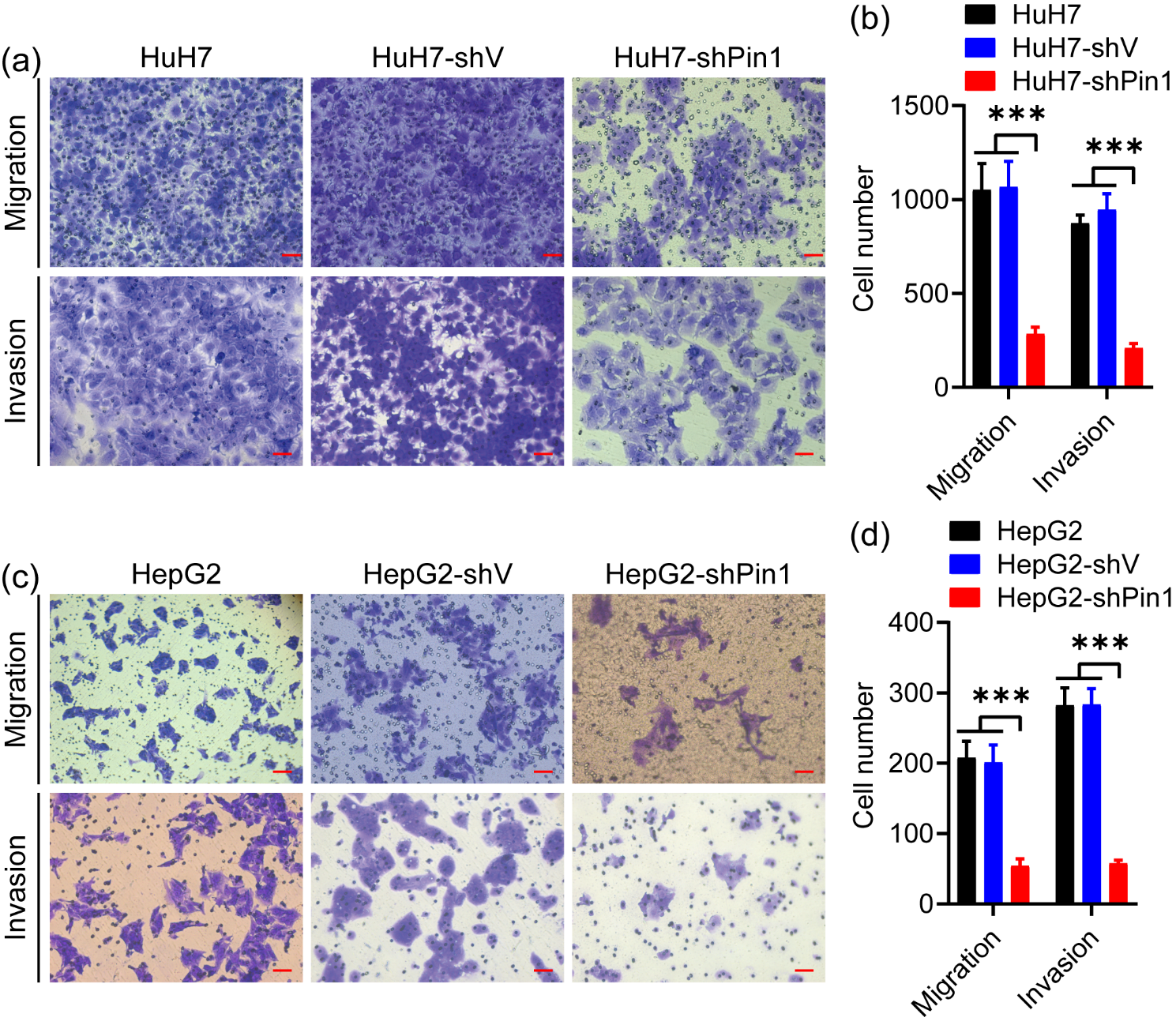


Figure S5. Effects of Pin1 knockdown on migration and invasion of liver cancer cells. (a) Images, and (b) numbers of migrated and invaded HuH7, HuH7-shV, and HuH7-shPin1 cells (mean ± SD, n = 3). (c) Images, and (d) numbers of migrated and invaded HepG2, HepG2-shV, and HepG2-shPin1 cells (mean ± SD, n = 3). *** *p* < 0.001.


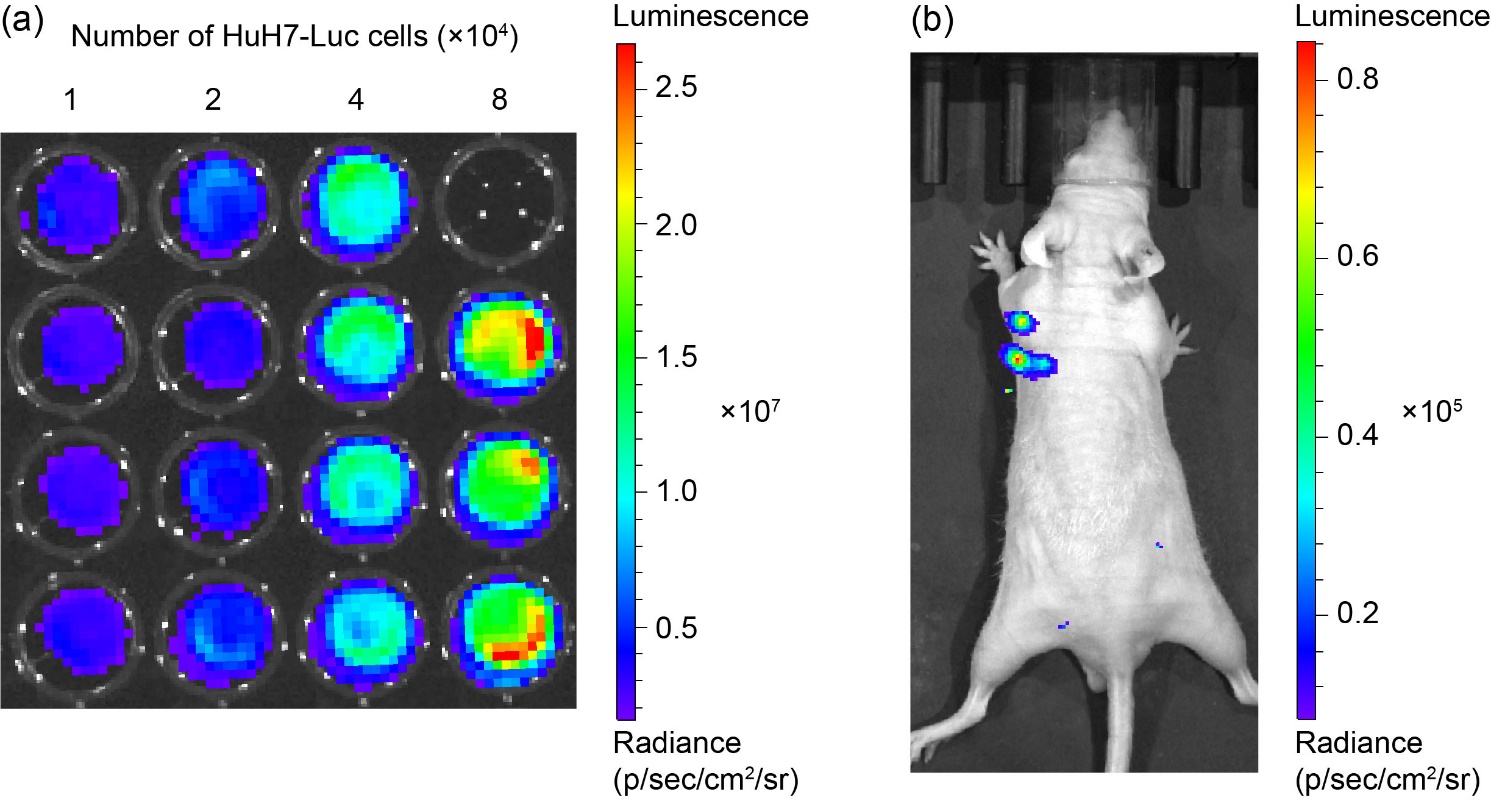


Figure S6. (a) *In vitro* bioluminescence imaging of HuH7-Luc cells. (b) Representative *in vivo* bioluminescence imaging of mice on day 7 after injection of HuH7-Luc cells.


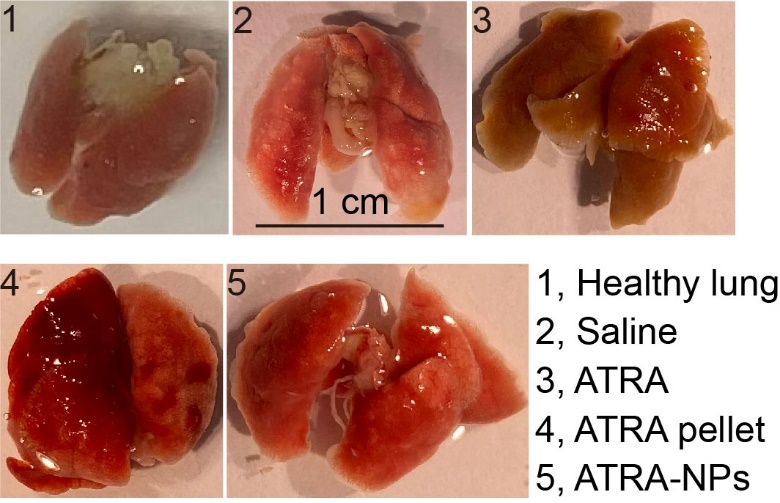


Figure S7. Representative photos of the lungs of mice after different treatments.


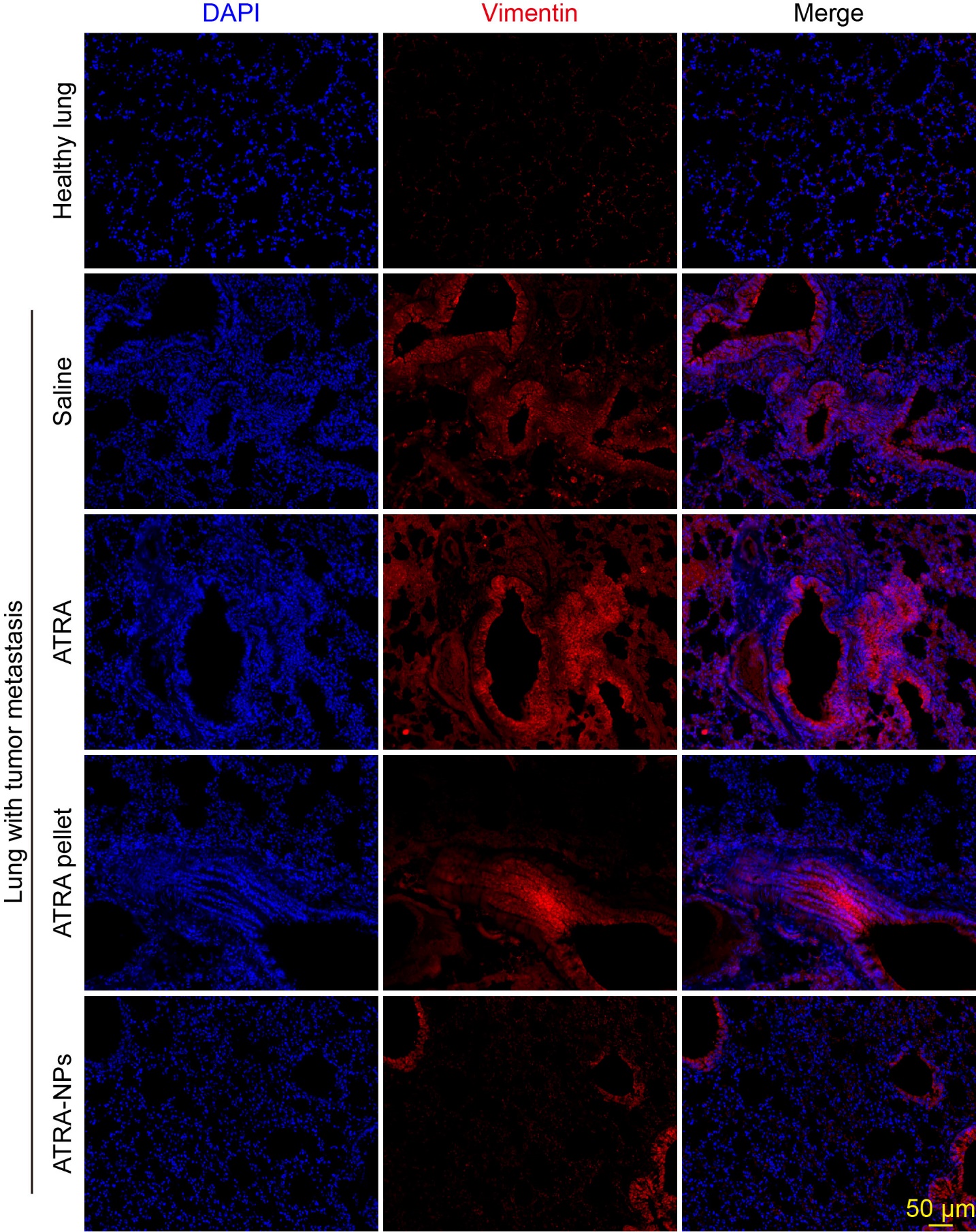


Figure S8. Immunofluorescence images of Vimentin in the lungs of mice after different treatments.


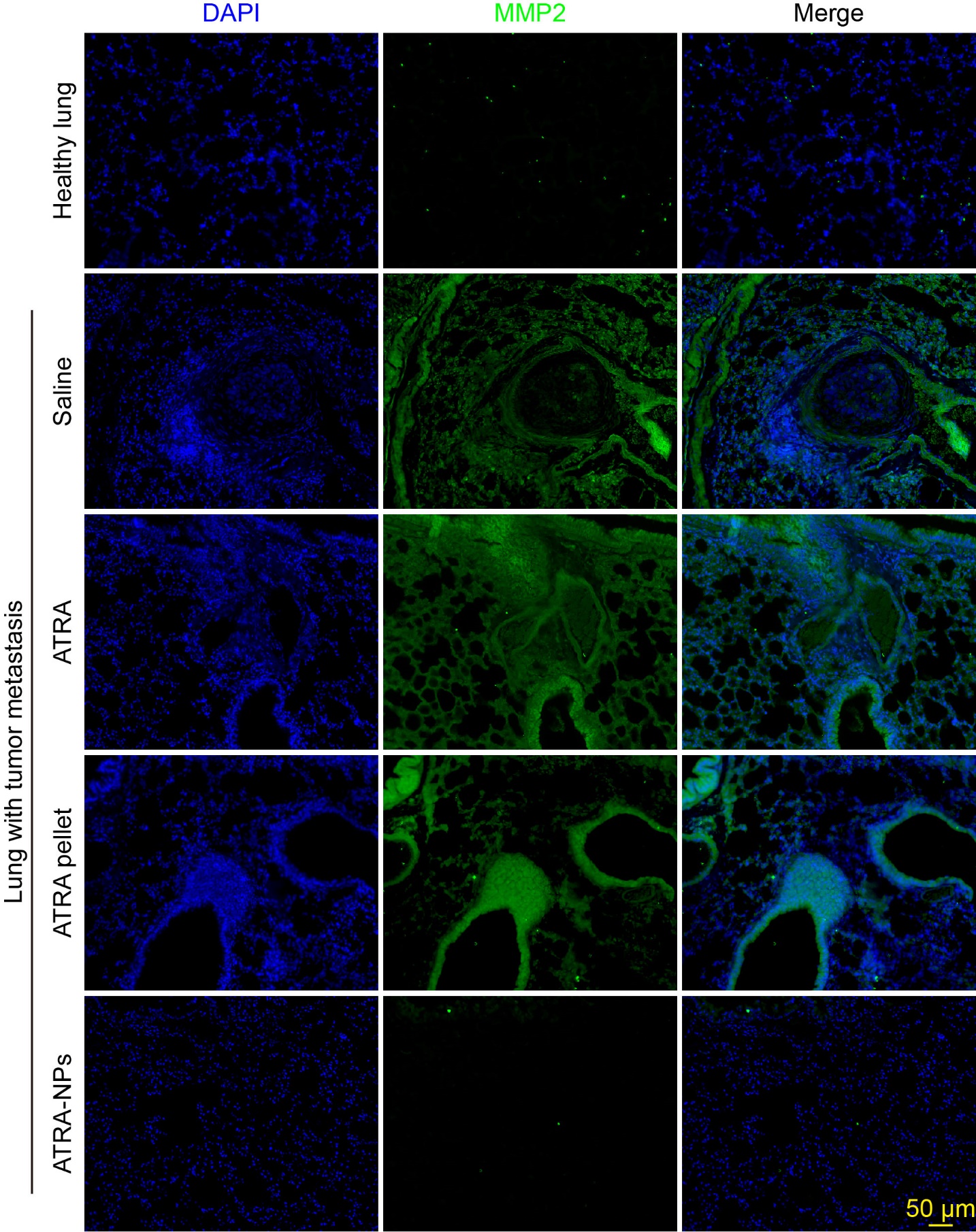


Figure S9. Immunofluorescence images of MMP2 in the lungs of mice after different treatments.


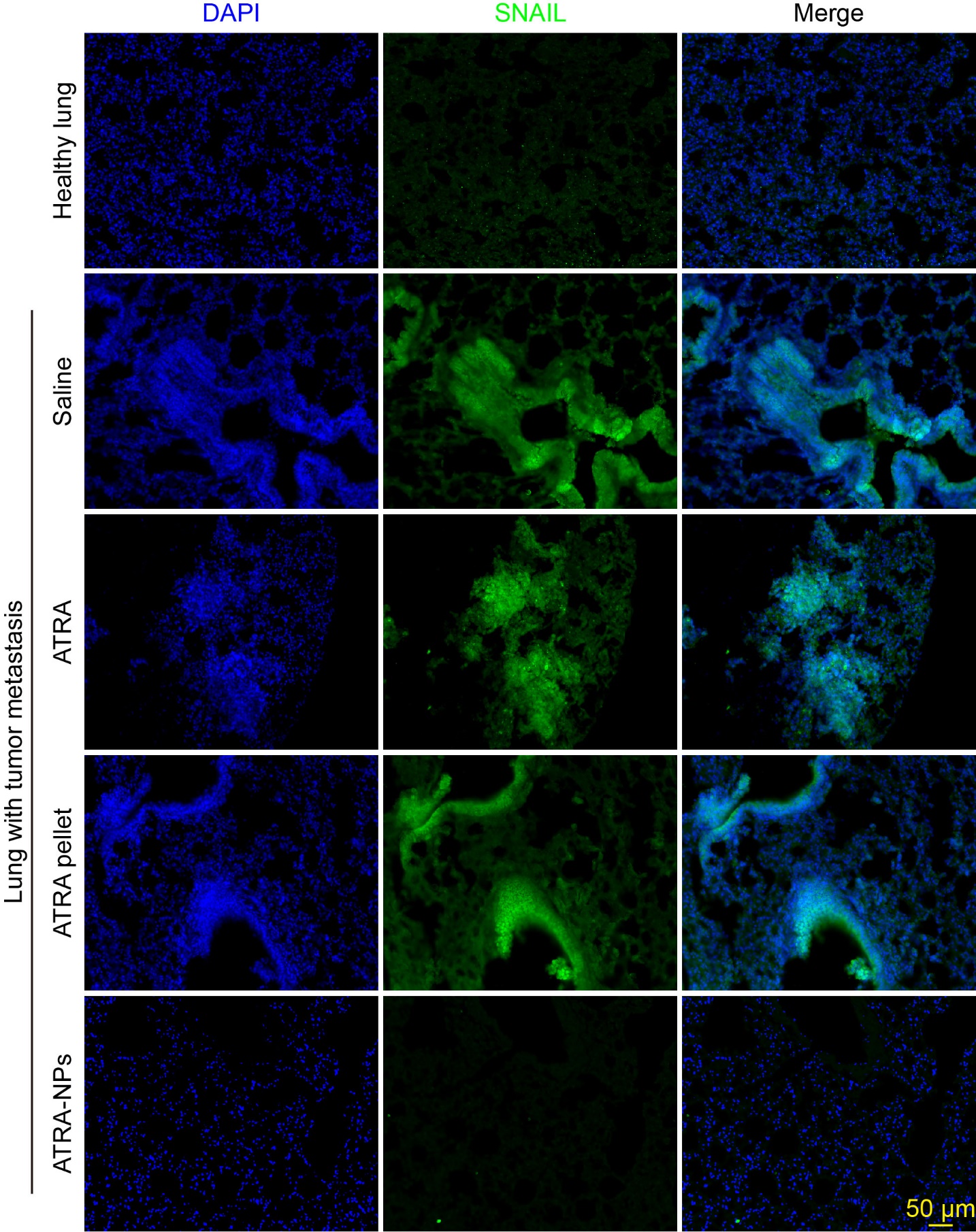


Figure S10. Immunofluorescence images of SNAIL in the lungs of mice after different treatments.


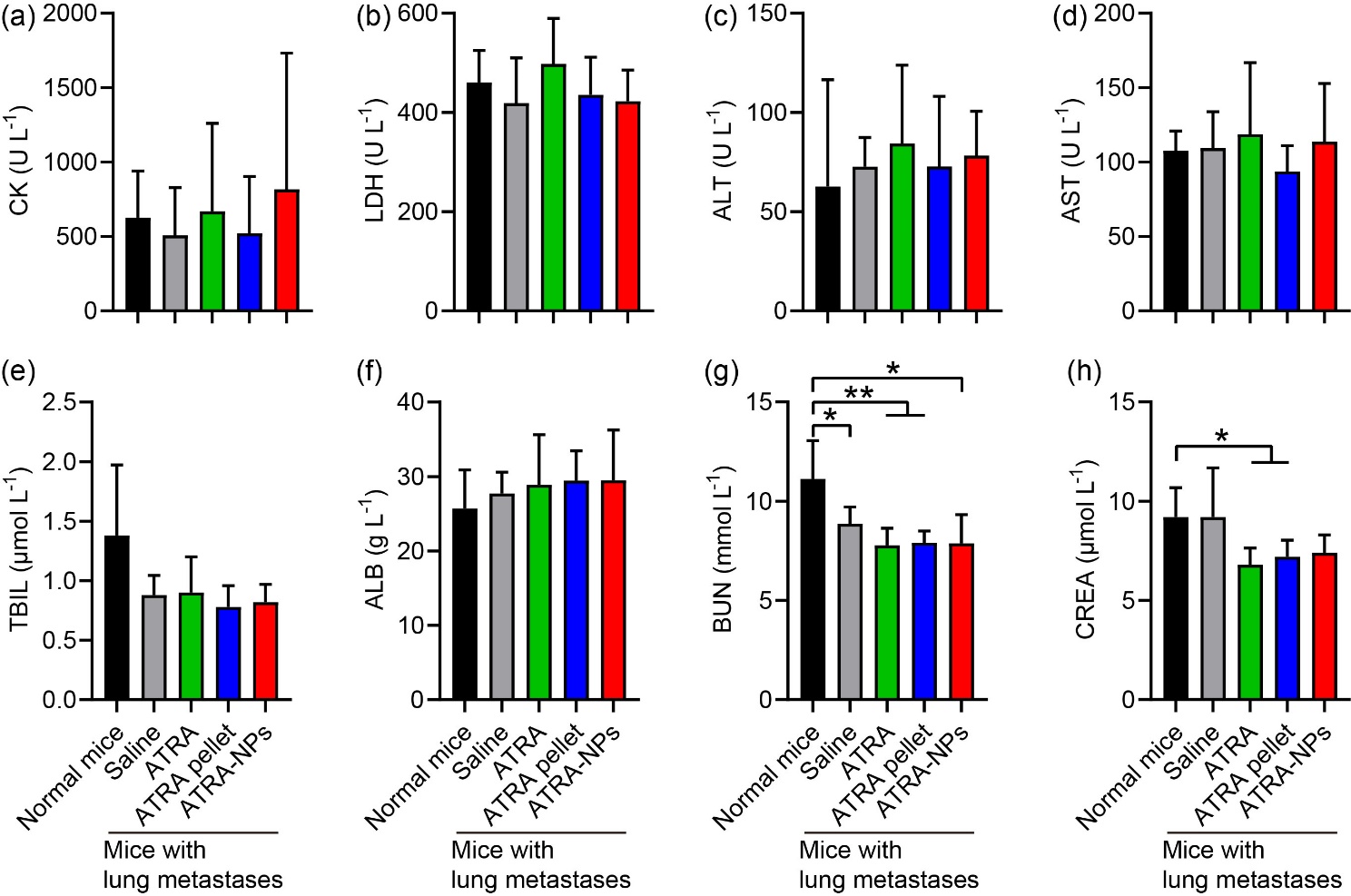


Figure S11. (a-h) The levels of functional indices of heart, liver and kidney in the plasma of mice after different treatments (mean ± SD, n = 5). CK: Creatine kinase; LDH: Lactate dehydrogenase; ALT: Alanine aminotransferase; AST: Aspartate aminotransferase; TBIL: Total bilirubin; ALB: Serum albumin; BUN: Blood urea nitrogen; CREA: Creatinine. * *p* < 0.05, ** *p* < 0.01.


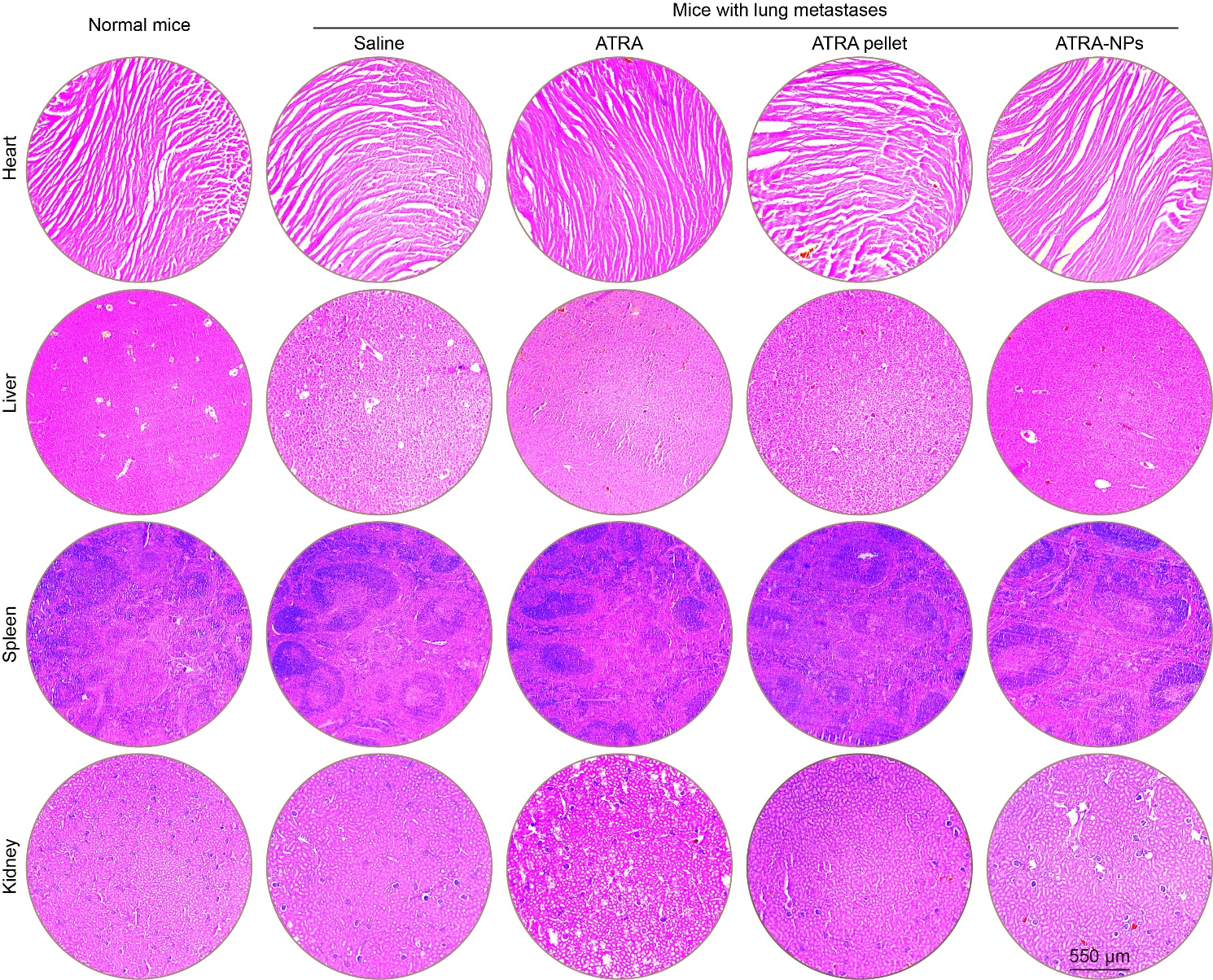


Figure S12. H&E staining photos of tissue sections of heart, liver, spleen and kidney of mice after different treatments.

1. Correspondence address. E-mail: dyyang@fjmu.edu.cn (D. Y. Yang); Tel: +86-591-22862498

   ^1^ These authors contributed equally to this work. [↑](#footnote-ref-1)
